# Transcription Factor‐Based Gene Therapy Enables Functional Repair of Rat Following Chronic Ischemic Stroke

**DOI:** 10.1111/cns.70448

**Published:** 2025-05-22

**Authors:** Tao Wang, Xu Wang, Shanggong Liu, Menglei Li, Kaiying Wan, Jiajun Zheng, Kai Liao, Jinyu Wang, Kaiming Zou, Lu Wang, Hao Xu, Wenliang Lei, Gong Chen, Wen Li

**Affiliations:** ^1^ Guangdong‐Hong Kong‐Macau Institute of CNS Regeneration (GHMICR) Jinan University Guangzhou China; ^2^ Key Laboratory of CNS Regeneration (Ministry of Education), Guangdong Key Laboratory of Non‐Human Primate Research, GHM Institute of CNS Regeneration Jinan University Guangzhou China; ^3^ State Key Laboratory of Bioactive Molecules and Druggability Assessment, Guangdong Basic Research Center of Excellence for Natural Bioactive Molecules and Discovery of Innovative Drugs Jinan University Guangzhou China; ^4^ Department of Nuclear Medicine and PET/CT‐MRI Center The First Affiliated Hospital of Jinan University & Institute of Molecular and Functional Imaging, Jinan University Guangzhou China

**Keywords:** astrocyte‐to‐neuron conversion, chronic phase, ischemic stroke, neural transcription factor, striatum, white matter repair

## Abstract

**Objective:**

In vivo transcription factor (TF) ‐mediated gene therapy through astrocyte‐to‐neuron (AtN) conversion has shown therapeutic effects on rodent and non‐human primate cortical ischemic injury in the subacute phase. However, in the clinic, subcortical regions including striatum as well as white matter are vulnerable regions of stroke, with millions of patients beyond subacute phase. In this study, we investigate whether TF‐mediated AtN conversion therapy can be extended to treat chronic‐phase ischemic stroke involving subcortical regions (e.g., striatum) and white matter, beyond cortical injuries.

**Methods:**

Rat middle cerebral artery occlusion (MCAO)‐like models were established to induce broad ischemic injuries including cortical and striatal regions. Then multiple rounds of TF‐mediated gene therapy treatments through adeno‐associated virus (AAV) system to cover the large‐scaled infarct areas were conducted in the chronic phase of the stroke models. Magnetic resonance imaging (MRI), [^18^F] FDG‐PET/CT, behavioral tests, immunohistochemistry and bulk‐RNA seq were applied to evaluate the AtN conversion, tissue repair and functional recovery.

**Results:**

Our results revealed that administrated in the chronic phase of ischemic stroke, TF‐mediated gene therapy can efficiently regenerate new neurons in both cortical and striatal regions, and promote tissue repair in both grey and white matter. Compared with single round of AAV administration, multiple rounds of treatment regenerated more neurons and led to a significant functional recovery.

**Conclusions:**

Our study demonstrates that TF‐mediated gene therapy has a broad therapeutic time window and can be applied multiple rounds to treat severe ischemic stroke, making it an attractive therapeutic intervention in the chronic phase after stroke, when current approaches are largely ineffective.

## Graphical abstract

Multiple rounds of transcription factor‐based gene therapy can efficiently regenerate neurons in a large‐scale rat ischemic stroke model in the chronic phase, rescue tissue atrophy, reduce scar formation, promote the recovery of neurovascular units, repair the white matter, and enhance functional recovery of the brain.

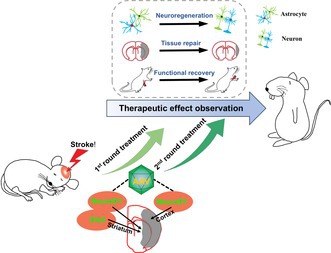

## 1 Introduction

Stroke is the second leading cause of death and the third leading cause of adult disability globally, leading to the damage of brain structure and impairment of functions including motion, sensory, language and cognition [1, 2, 3]. Systemic thrombolysis and mechanical thrombectomy are two clinically approved treatments for acute ischemic stroke, but are only suitable for a small number of patients due to time constraints and restrictive selection criteria [4]. Massive neuronal death is an important pathological feature of stroke [5], thus regenerating new neurons to replenish the lost neurons is critical to the treatment of stroke. Unfortunately, the adult mammalian brain has a very limited capacity to regenerate sufficient neurons to repair the damaged tissue [6, 7]. Transplantation of external neural progenitor cells (NPCs) has been explored as a potential stroke therapy, but faces serious challenges such as immunorejection, tumorigenesis, and long‐term survival of transplanted cells [8, 9]. Alternatively, the endogenous glial cells including astrocytes and oligodendrocyte precursor cells can divide after injury and spontaneously transdifferentiate to very limited number of neuroblasts under certain circumstance [10, 11]. Such latent neurogenic capacity of the endogenous glial cells may be augmented through the ectopically expression of neural transcription factors (TFs) including NEUROD1, SOX2, NGN2, ASCL1 and DLX2, or combinations of neural TFs ASCL1+LMX1A+NURR1, NEUROD1+DLX2, and NGN2+NURR1 [12, 13, 14, 15, 16]. This in vivo glia‐to‐neuron conversion sheds light on its potential applications in regenerative medicine and has shown therapeutic effects on a number of animal models of neurological disorders, including Parkinson' disease, ischemic stroke, epilepsy, Huntington's disease and spinal cord injury [17, 18, 19, 20, 21, 22, 23].

Previous studies applied the NEUROD1‐mediated astrocyte‐to‐neuron (AtN) conversion approach to focal ischemic injury models restricted to cortical regions. When NEUROD1 was ectopically expressed in the reactive astrocytes around the ischemic cortical areas through AAV or lentiviral delivery system, it efficiently regenerated new neurons, repaired cortical tissues and promoted functional recovery of the injured mice [19, 24, 25, 26]. In addition to the mouse ischemic models, NEUROD1 also efficiently converted reactive astrocytes into neurons in the non‐human primate ischemic cortex, and repaired the damaged cortical tissues [18]. These results suggest the in vivo NEUROD1‐mediated AtN conversion as a promising intervention to treat ischemic stroke. Nevertheless, for better clinical translation, several challenges need to be tackled. For example, the ischemic regions in the previous studies are confined to a relatively small cortical region, but clinically relevant ischemic strokes often occur in subcortical regions including striatum and white matter [27]. In addition, the intervention time selected in the previous studies is the early stage of the stroke models (from 3 to 10 days in mouse models and 21 days in primate models) [18, 19]. It is worthwhile to investigate whether AtN conversion still has therapeutic effects when applied in the relatively chronic phase (30 days or later) after the ischemic insult in multiple brain regions including striatum and white matter.

## 2 Materials and Methods

### 2.1 Endothelin‐1 Induced MCAO in Rat

All efforts were made to minimize animal suffering and to reduce the number of animals used. Adult Sprague Dawley rats (male and female, over 8‐week‐old, weighting 250–300 g) were housed at a constant temperature of 22°C ± 2°C maintained on a 12–12 h light–dark cycle (07:00–19:00) and provided with food and water ad libitum. Animals were anesthetized with isoflurane (1.5%–2% in air). Temperature was maintained at 37°C throughout the surgery using a self‐regulating heating blanket. To monitor the change in cerebral blood flow during and after the occlusion, a laser probe of laser doppler flowmetry (moorVMS‐LDF SN480) was placed at the border‐zone territory between ACA and MCA territory (AP: +2 mm, ML: +2 mm). Endothelin‐1 (ET‐1, GL biochem, Shanghai) was dissolved in sterile saline to 0.2 μg/μL and delivered near the rat middle cerebral artery (MCA) via stereotaxic injection as previously described with minor modification [28, 29]. Briefly, in order to cover the MCA as much as possible, A total of 5 μL ET‐1 was injected at two depths (AP: +0.9 mm, ML: +5.2 mm, DV: −8.1/−8.3 mm) at the speed of 0.5 μL/min through a micro infusion pump. The needle was left in situ for 8 min after the injection before being slowly removed. After the surgery, the animals were resuscitated on a heated mat at 37°C and returned to a clean cage after full recovery. All animal procedures were approved by the Animal Care and Use Committee at Jinan University with the approval IACUC‐20220905‐11.

### 2.2 Triphenyl Tetrazolium Chloride (TTC) Staining

48 h after the occlusion, animals were euthanized. The brains were immediately harvested and cut into 2 mm thick brain slices. The slices were then incubated with 2% TTC solution in a 37°C incubator in the dark for 15 min. The brain slices were then transferred to 4% PFA for 24‐h fixation, and finally photographed for recording.

### 2.3 AAV Virus Production

Single stranded adenovirus‐associated viral (ssAAV) vectors *GFAP104::GFP*, *GFAP104::NeuroD1‐P2A‐GFP* and *GFAP104::Dlx2‐P2A‐GFP* were used in this paper. The viral vectors were constructed and AAV serotype 9 (AAV9) was produced by PackGene Biotech LLC (Guangzhou, China). Iodixanol gradient (Merck/Sigma‐Aldrich, St Louis, MO, USA) ultracentrifugation was used for AAV purification. Virus concentrations were adjusted to 1 × 10^12^ GC/mL in 0.001% Pluronic F‐68 solution (Poloxamer 188 Solution, PFL01‐100ML, Caisson Laboratories, Smithfield, UT, USA) for intraparenchymal injection.

### 2.4 Stereotaxic Viral Injection

The coordinates of virus injection were determined one animal by one animal based on the MRI‐T2 images displaying the injured regions (Figure S1A). From the MRI, we calculated the length of the lesion from the rostral to the caudal side, then injected two sites evenly covering the edge of injured cortical regions (AAV9‐*GFAP104::NeuroD1‐P2A‐GFP*, 1uL/site,100 nL/min) and one site at the edge of injured striatal regions (AAV9‐*GFAP104::NeuroD1‐P2A‐GFP + AAV9‐GFAP104::Dlx2‐P2A‐GFP*, 2 uL/site, 200 nL/min). The exact coordinates will be determined according to the injury of each rat by combining MRI and rat brain atlas. After the injection, the needle was left in situ for 8 min before being slowly removed. The second round of injections was performed in the same position with the same recipes and dosage as the first round. After the operation, the rats were placed in a comfortable cage for resuscitation.

### 2.5 Immunohistochemistry and Analysis

The rats were anesthetized with 2% pentobarbital sodium (0.2 mL/100 g) and sequentially perfused with normal saline followed by 4% paraformaldehyde (PFA) in PBS to fix the brain. The brains were collected and postfixed in 4% PFA overnight and sequentially placed in 10%, 20%, 30% sucrose at 4°C until the tissue sank. Then embed the brain with Optimal Cutting Temperature (Tissue‐Tek O.C.T. Compound; Sakura Finetek, Torrance, CA, USA); the brain was serially sectioned at the coronal plane on the cryostat (Thermo Scientific, Shanghai, China) at 60 μm thickness. For immunofluorescence, brain slices were washed three times in PBS for 10 min each and then blocked in the buffer solution (5% Donkey serum, 0.3% TritonX‐100, 3% Bovine Serum Albumin) for 1 h at room temperature. Primary antibodies were diluted in the blocking buffer and incubated at 4°C for 24 h. Then the brain slices were washed three times with 0.2% PBST (0.2% tween‐20 in PBS) and incubated with DAPI (4′,6‐diamidino‐2‐phenylin‐dole) and the secondary antibodies conjugated to Alexa Fluor 488, Alexa Fluor 555, or Alexa 539 Fluor 647 (1:1000) for 2 h at room temperature, followed by extensive washing with PBS. The samples were finally mounted on the glass and sealed with nail polish. Images were acquired with ZEN software (Zeiss) on AXIO Imager Z2 (Zeiss) and LSM880 confocal microscope (Zeiss). The images were analyzed by Zeiss software ZEN and ImageJ software. The detailed information of antibodies used in this study is listed in Table S1.

### 2.6 Whole‐Cell Patch‐Clamp Recordings

Brain slices were prepared typically 60 days after 1‐round virus injection and cut at 300 μm thickness on the horizontal plane with a Leica vibratome (VT‐1200S) in ice‐cold cutting solution (containing 87 mM NaCl, 75 mM sucrose, 0.5 mM CaCl_2_, 2.5 mM KCl, 4 mM MgCl_2_, 1.25 mM NaH_2_PO_4_, 24 mM NaHCO_3_, and 25 mM glucose). Slices were incubated in NMDG‐ACSF (containing 93 mM NMDG, 1.2 mM NaH_2_PO_4_, 2.5 mM KCl, 30 mM NaHCO_3_, 25 mM glucose, 20 mM HEPES, 5 mM sodium ascorbate, 3 mM sodium pyruvate, 2 mM Thiourea, 10 mM MgSO_4_·7H_2_O, 0.5 mM CaCl_2_, pH 7.3 adjusted with HCl, 300–310 mOsm/L), continuously bubbled with 95% O_2_ and 5% CO_2_, first at 34°C for 30 min, and then at room temperature. Whole‐cell recordings were performed using a Multiclamp 700B patch‐clamp amplifier (Molecular Devices, Palo Alto, CA), and the slices were maintained in artificial cerebral spinal fluid (ACSF) containing 126 mM NaCl, 2.5 mM KCl, 1.25 mM NaH_2_PO_4_, 26 mM NaHCO_3_, 2 mM MgCl_2_, 2 mM CaCl_2_, and 10 mM glucose. The pH of the bath solution was adjusted to 7.3 with NaOH, and osmolarity at 310–320 mOsm/L. Patch pipettes were pulled from borosilicate glass (~5–8 MΩ) and filled with a pipette solution consisting of 126 mM K‐Gluconate, 4 mM KCl, 10 mM HEPES, 4 mM Mg_2_ATP, 0.3 mM Na_2_GTP, 10 mM Phospho‐Creatinine (pH 7.3 adjusted with KOH,290 mOsm/L). Data were collected using pClamp 10 and Clampex 10.4 software (Molecular Devices, Palo Alto, CA), sampled at 10 kHz and filtered at 3 kHz, analyzed with Clampfit 10.4.

### 2.7 Magnetic Resonance Imaging (MRI) and Infarct Volume Measurement

MRI was performed using a high‐field small animal magnetic resonance system with a magnetic field strength of 9.4 Tesla and a magnet diameter of 30 cm (Bruker Biospec 94/30USR). T2‐weighted images to visualize infarction were acquired using MSME sequences. The scanning parameters of T2‐weighted imaging were as follows: TE 33.00 ms, TR 3500.00 ms, a total of 31 slices, and the scanning range included the whole brain from the olfactory bulb to the cerebellum. Data were analyzed using RadiAnt DICOM Viewer software for image localization, and injury volume statistics were analyzed using Itk‐snap for 3D analysis and volume statistics. DTI (Diffusion Tensor Imaging) can quantitatively evaluate the anisotropy of the brain white matter and thus evaluate the condition of the white matter. The DTI scanning parameters were as follows: TE 20.70 ms, TR 3000.00 ms, and the scanning range included the whole brain from the olfactory bulb to the cerebellum. Dsi‐studio was used for DTI data analysis, fiber tracts reconstruction, measurement of fractional anisotropy (FA), axial diffusion (AD), radial diffusion (RA), mean diffusion (MD) values, and 3D structure reconstruction.

### 2.8 [ ^18^F] FDG‐PET/CT[MOU1] Imaging

PET/CT imaging experiments were performed on an IRIS small animal PET/CT imaging system (inviscan SAS, Strasbourg, France). [^18^F] fluorodeoxyglucose (FDG:0.6 mCi in 500 μL) to investigate glucose metabolism was injected through a tail vein. Emission data acquisition was 1 h starting with tracer injection. PET reconstruction was performed using the 3D ordered subset expectation maximum algorithm based on a Monte Carlo accurate detector model, and image voxels were 0.855 × 0.855 × 0.855 mm^3^. The FDK algorithm was used for CT reconstruction, and the image voxels were 0.16 × 0.16 × 0.16 mm^3^.

### 2.9 Adhesive Removal Test

An adhesive removal test to evaluate sensorimotor asymmetries was performed as described [30, 31]. Briefly, a sleeve was created using a 3.0 cm piece of green paper tape (1.0 cm in width) and wrapped around the injured forepaw so that the tape attaches to itself and that the fingers protrude slightly from the sleeve formed. The typical response of the rat with the attached tape is that the animal vigorously attempts to remove the sleeve by either pulling the tape with its mouth and/or brushing the tape with its contralateral paw. The rat was then placed in its cage and observed for 1 min. Two timers were set during the test: the first timer was kept running without interruption, whereas the other was turned on only when the animal attempted to remove the tape sleeve. The cumulative time of contacting the adhesive stickers within 1 min is used to evaluate the sensory ability of the animal (Touching time). The test was repeated three times per testing day and the best two scores were averaged.

### 2.10 Cylinder Test

Cylinder test to evaluate the forepaw asymmetry was performed as previously reported [32]. The rats were individually placed in a transparent cylinder with a diameter of 20 cm and a height of 30 cm for 5 min. A mirror was placed left behind the cylinder to reduce the observation blind area of the experiment. The use of both forelimbs was observed during exploratory activities. Forelimb use was measured during vertical exploration. Each forepaw contact with the cylinder wall was counted. When simultaneous limb contact was observed, a touch was counted for each paw. The asymmetry score of forelimbs use in wall exploration was calculated for each group (injured forelimb/(injured + contralateral)) to obtain a score where 0.5 represents perfect symmetry and any number closer to zero would suggest a decrease in the use of the injured limb.

### 2.11 Bulk RNA Seq

Ten days (10D) and sixty days (2R 30 + 30D) after virus injection, rats were anesthetized with isoflurane and perfused with ice‐cold artificial cerebrospinal fluid. Then the virus infected brain regions were harvested for bulk mRNA‐seq and analysis. RNA extraction was done by Gene De novo Biotechnology (Guangzhou, China) and RNA purity and concentration were assessed using NanoDrop 2000 Spectrophotometer detection. The sequencing was performed on the Illumina HiseqTM 2500/4000 by Gene DenovoBiotechnology. RNA differential expression analysis was performed using DESeq2 software between two different groups. The genes/transcripts with the parameter *p* value < 0.05 and absolute fold change ≥ 2 were considered differentially expressed genes/transcripts. GO, KEGG, and GSEA analysis were performed using Omicsmart, a real time interactive online platform for data analysis (http://www.omicsmart.com). GO enrichment analysis provides all GO terms significantly enriched in DEGs compared with the genome background, and filters the DEGs that correspond to biological functions. First, all DEGs were mapped to GO terms in the GO database, and gene numbers were calculated for every term. Significantly enriched GO terms in DEGs compared with the genome background were defined by hypergeometric test.

Pathway‐based analysis helps to further understand genes' biological functions. KEGG is the major public pathway‐related database. Pathway enrichment analysis identified significantly enriched metabolic pathways or signal transduction pathways in DEGs compared with the whole‐genome background.

### 2.12 Statistical Analysis

The numbers of the interested cells were counted with ImageJ 1.51j8 from the pictures of 8–10 randomly chosen fields with the thickness of 20 μm taken with a confocal microscope (212.55 μm × 212.55 μm, LSM880 confocal microscope, Zeiss), from 3 to 5 consecutive sections from each animal. Five to six animals were included in each group.

Fluorescence intensity of the region of interest was measured from pictures from 3 to 5 serial brain sections from each rat using Zeiss software ZEN, which was normalized to the fluorescence intensity of the lateral ventricle. Five to six animals were included in each group.

The lengths of the vessels were analyzed using the Angiogenesis Analyzer plugin of Image J from the images acquired by a confocal microscope (212.55 μm × 212.55 μm, LSM880 confocal microscope, Zeiss). 3–5 brain slices were analyzed for each rat, 8–10 points were taken from each brain slice striatum, and the average value was taken as the representative vessel length.

All results were expressed as Mean ± SD. Statistical analysis was performed using one‐way ANOVA or two‐way ANOVA for multiple groups, and paired or unpaired t‐test for two groups (Prism7; GraphPad Software Inc., La Jolla, CA). The detailed information of *p* values and statistical tests was elaborated in figures and figure legends. *p* < 0.05 was considered statistically significant.

## 3 Results

### 3.1 Establishment of Endothelin‐1 Induced MCAO Model in Rats

**FIGURE 1:**
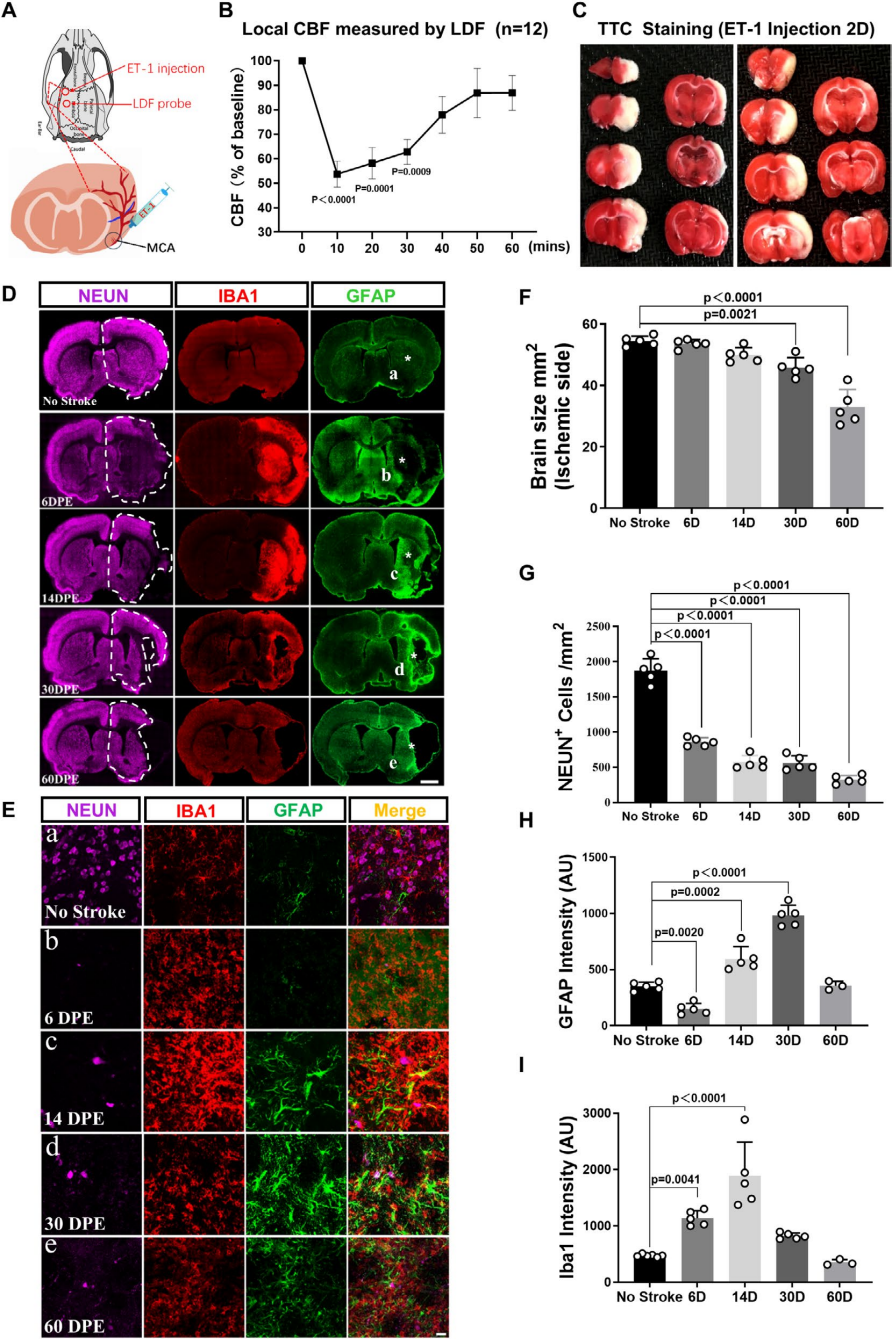
Establishment of endothelin‐1 induced MCAO‐like model in rat. (A) The schematic shows the injection site of endothin‐1 (1–31, ET‐1) and the placement of the Laser Doppler Flowmeter probe. (B) Quantification showing the change of blood flow after the ET‐1 injection. Note that Blood flow decreased significantly ten minutes after ET‐1 injection and gradually returned to normal levels within an hour (One‐way ANOVA followed by Tukey's post hoc test; *n* = 12 animals). (C) TTC staining showing the ischemic infarcts in the cerebral cortex and striatum two days after ET‐1 injection. Note the white areas indicating the ischemic regions including cortical and striatal regions. (D) Typical images illustrating the pathogenesis at different time points after ET‐1 induced ischemic injury (6, 14, 30, and 60 days post ET‐1 injection, DPE; normal brains/no stroke were used as negative normal control). The white dashed lines delineate the ischemic injured hemispheres. Neurons are labeled by NEUN in magenta. Microglia is labeled by IBA1 in red. (GFAP) astrocytes are labeled by GFAP in green. Scale bar = 2000 μm. (E) High magnification imaging showing the regions marked by white asterisks in (D). Note that GFAP signal in the ischemic injured striatum lost at 6 DPE (b), and reappeared later at 14 DPE (c) and 30 DPE (d) and 60 DPE (e). Scale bar = 20 μm. (F–I) Quantifications of remaining hemispheres (F), neuronal density (G), and activation of astrocytes (H) and microglia (I) at 6, 14, 30, and 60 days after ET‐1 injection (One‐way ANOVA followed by Tukey's post hoc test; *n* = 5 animals for each group).

In order to establish a more severe ischemic stroke model embracing subcortical regions including the striatum, we injected endothelin‐1 (ET‐1, 1–31), a blood vessel constricting factor, to the vicinity of the rat middle cerebral artery to establish a MCAO (middle cerebral artery occlusion)‐like ischemic stroke model as previously described [33]. Injection of ET‐1 induced a 50% decrease in the blood flow downstream of the middle cerebral artery 10 min after the injection, revealed by the laser Doppler flowmeter (LDF) (Figure 1A,B). Within the subsequent 1 h, the blood flow recovered to the original level, suggesting a reperfusion (Figure 1B). Two days later, triphenyltetrazolium chloride (TTC) staining revealed a large infarct area including both cortical and striatal regions in the injection side (Figure 1C), mimicking the classical suture‐induced MCAO model [34, 35]. This ET‐1 induced MCAO‐like model utilizes a simplified injection procedure instead of complicated blood vessel isolation and suture procedures, which often lead to high mortality. To characterize the pathological progression of the ET‐1 MCAO model, we performed a series of immunostaining to examine both neuronal and glial changes at different time points after ET‐1 injection (Figure 1D). Specifically, NEUN staining (magenta) showed progressive neuronal loss from 6 to 60 days post ET‐1 injection (DPE), accompanied by activation of both microglia (IBA1, red) and astrocytes (GFAP, green) (Figure 1D). Enlarged images in Figure 1E better illustrate the reactive microglia and reactive astrocytes in the center area of the striatum after stroke. Overall, a significant tissue loss in the injured cortex and striatum was detected at 30 DPE and continued to deteriorate at 60 DPE (Figure 1F). Quantitative analyses revealed that the neuronal density in the injured hemisphere showed a significant decrease at 6 days after injury (6 DPE) and continued the decreasing trend by 60 DPE (Figure 1G). In contrast, the intensity of GFAP, an indicator of the reactivity of astrocytes, peaked at 30 DPE and then decreased to the normal range by 60 DPE (Figure 1H). Different from the astrocytes, microglial activation (IBA1) increased much earlier and peaked at 14 DPE, followed by a significant decrease at 30 DPE (Figure 1I). These results indicated that the ET‐1‐induced MCAO model shared many pathological features with the traditional transient MCAO model [36, 37]. Since 30 days after the ischemic insult is considered the chronic phase in traditional rodent MCAO models, and the reactivity of astrocytes in this MCAO‐like model peaked at 30 DPE, we chose 30 DPE as the time point for therapeutic intervention.

### 3.2 Enhanced Neural Regeneration After Two Rounds of TF Treatment

**FIGURE 2:**
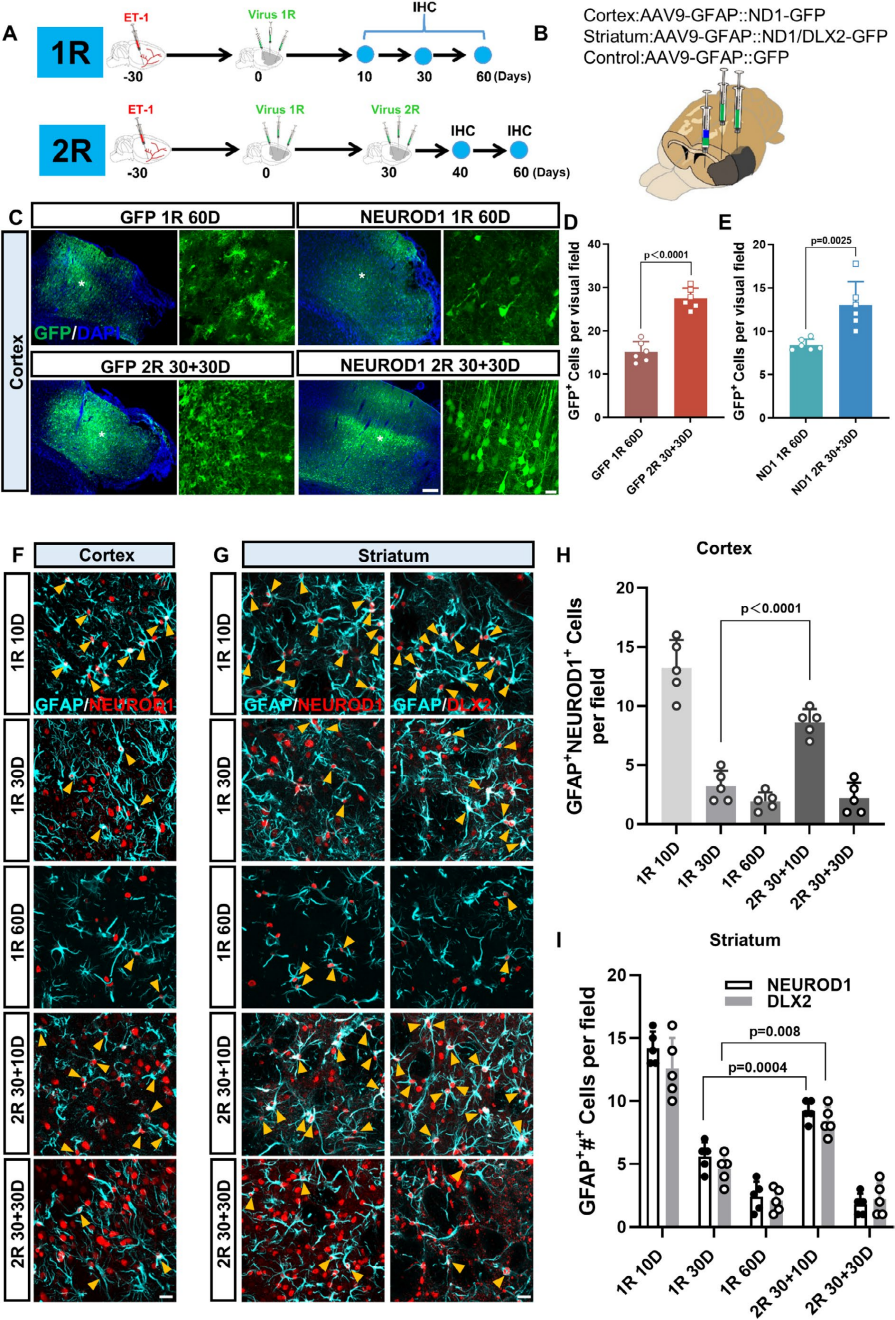
Two rounds of AAV administration increase the infection areas. (A) Schematic diagram showing the experimental design. 1R (1 round of AAV administration), 2R (2 rounds of AAV administration). (B) Schematic showing the locations to inject the viruses. The black area of the brain indicates the infarct region. AAV‐NeuroD1 was injected to the injured cortex, and AAV‐NeuroD1 + Dlx2 was injected to the injured striatum. AAV‐GFP was used as the control. (C) Representative pictures illustrating the virus spread area in the injured cortical regions. Note the larger infection areas (indicated by GFP signal, green) after the 2 rounds of AAV administration. Enlarged views of the positions marked by white asterisks are listed on the right side of each block. Scale bars = 200 μm, 20 μm. (D, E) Quantification of the density of GFP positive cells in the control groups (D) and TFs groups (E). (Unpaired *t*‐test; *n* = 6 animals per group.) (F) Representative images showing the expression of NEUROD1 (Red) in astrocytes (GFAP, Cyan) at different time points in the cortex. The cells double positive for NEUROD1 and GFAP are marked by the yellow arrow heads. Scale bar = 20 μm. (G) Typical pictures showing the colocalization of NEUROD1 (red, left column) or DLX2 (red, right column) with GFAP (cyan) in the striatum at different time points. The cells double positive for either NEUROD1 and GFAP, or DLX2 and GFAP, are marked by the yellow arrow heads. Scale bar = 20 μm. (H) Quantification showing the number of astrocytes expressing NEUROD1 in the ischemic injured cortex at different time points. (One‐way ANOVA followed by Tukey's post hoc test; *n* = 5 animals for each group.) (I) Quantification illustrating the number of astrocytes expressing either NEUROD1 (white column) or DLX2 (gray column) in the ischemic injured striatum at different time points. (One‐way ANOVA followed by Tukey's post hoc test; *n* = 5 animals for each group).

After establishing a MCAO‐like stroke model, we sought to investigate a potential treatment for such severe stroke injury across the cortex and striatum. According to our previous studies, NEUROD1 alone is efficient in regenerating cortical neurons in the mouse cortex after focal ischemic injury [19], whereas NEUROD1 plus DLX2 can regenerate GABAergic neurons in the striatum in mouse models of HD [21]. Therefore, we selected to overexpress NEUROD1 in reactive astrocytes of the injured cortex (AAV9 *GFAP::Neurod1‐P2A‐GFP*) and overexpress NEUROD1 + DLX2 in the reactive astrocytes of the injured striatum (AAV9 GFAP::*Neurod1*‐P2A‐GFP + *GFAP::Dlx2‐P2A‐GFP*) at 30 days after injury (Figure 2A). Since the ischemic area in this MCAO‐like stroke model is quite large, we suspected whether one round of transcription factor (TF) treatment was sufficient to treat such severe injury, and hence explored whether two rounds of AAV treatments might have better repairing effects [18, 19, 21] (Figure 2A,B, Figure S1A). Indeed, we found that two rounds of AAV infection enhanced GFP signal area, suggesting enlarged infection areas in both the control and the TF groups (Figure S1B–D). Quantitative analysis revealed that two rounds of AAV injection not only enlarged the infection area but also increased the density of infected cells (GFP^+^ cells) (Figure 2C–E, Figure S1E–G). Immunostaining on TFs revealed that both NERUOD1 and DLX2 were initially expressed in GFAP^+^ astrocytes at 10 days post infection (Figure 2F,G). At 30 days post infection, the number of TF‐expressing astrocytes decreased both in the cortex and in the striatum (Figure 2H,I, 1R 30D), possibly due to some astrocytes converting into neurons (see below). Interestingly, at 10 days post 2nd round of TF‐infection, the number of TF‐expressing astrocytes increased again compared to that at 30 days after one round of TF‐infection (Figure 2F,G, 2R30 + 10D). Quantitative analyses revealed a decrease of TF‐expressing astrocytes from 10 to 30 days post one round of viral infection, followed by an increase after the 2nd round of viral infection (Figure 2H,I). The number of TF‐expressing astrocytes at 60 days post one round of viral infection (1R 60D) was comparable to that at 30 days post two rounds of viral infection (2R 30 + 30D), both at a very low level (Figure 2H,I). No transcription factor expression was detected in the control virus group (Figure S1H). The increased number of TF‐expressing astrocytes after the 2nd round of viral infection suggested that additional astrocytes were infected by the 2nd round of AAV injection.

**FIGURE 3:**
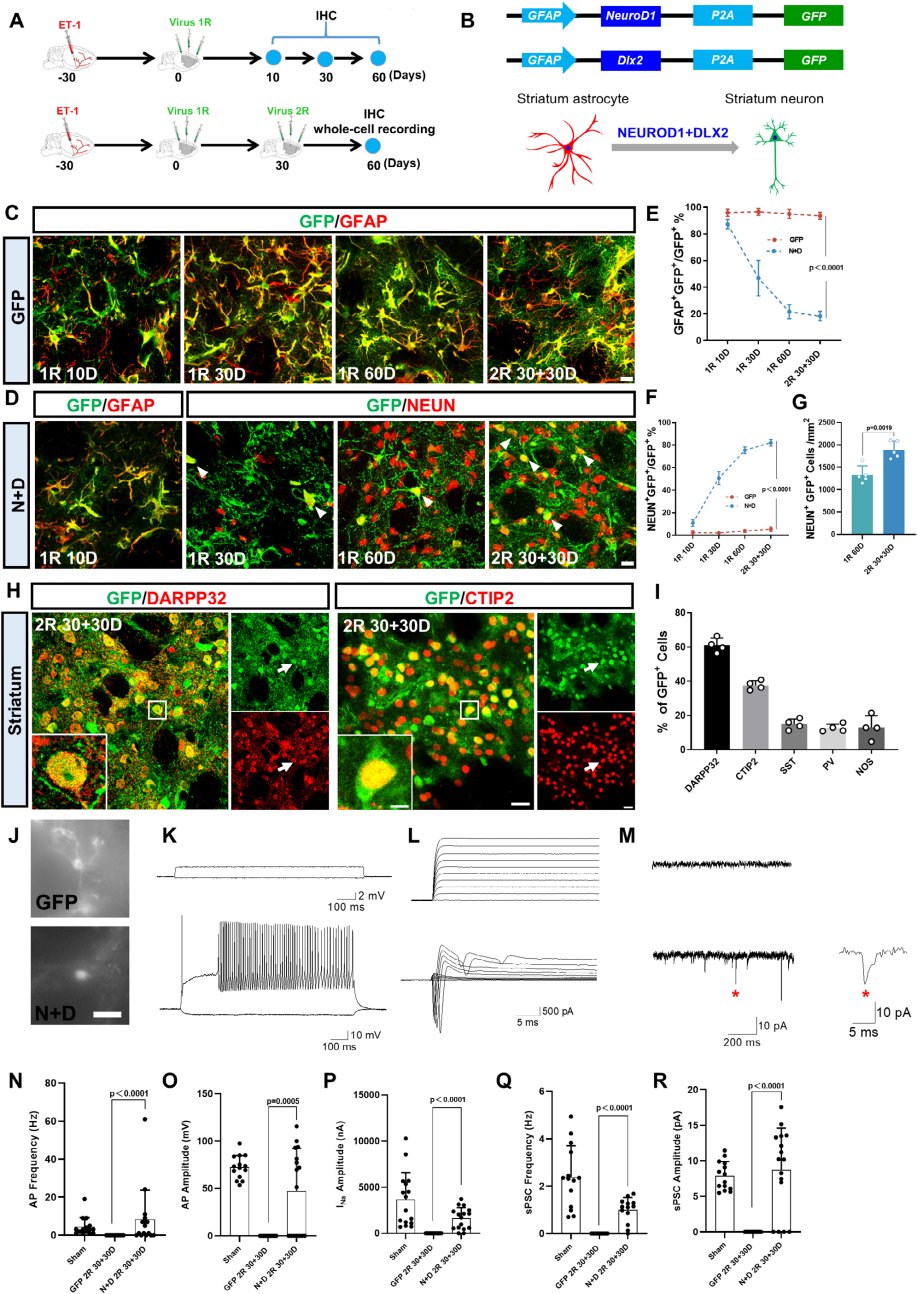
Two rounds of AAV‐mediated AtN conversion in striatum. (A) Schematic diagram showing the experimental design. (B) Diagram illustrating NEUROD1 + DLX2 induced AtN conversion process in the injured striatum. (C) The control virus infected cells (GFP, green) expressed astrocyte marker GFAP (red) at different time points. Scale bar = 20 μm. (D) Cells infected by NEUROD1 and DLX2 expressed astrocyte marker GFAP at 10D, but gradually expressed neuronal marker NEUN at later stages (1R 30D, 1R 60D, and 2R 30 + 30D). Scale bar = 20 μm. (E) Quantification showing that the percentage of GFP/GFAP double‐positive cells in *N* + D group (blue dashed line) continued to decrease over time, whereas the rate in the control virus group (red dashed line) remained at 95.2% ± 1.8% (Two‐way ANOVA followed by Sidak's multiple comparisons; *n* = 5 animals per group). (F) Quantification illustrating the percentage of GFP/NEUN double‐positive cells in *N* + D group gradually increased (blue dashed line) over time, but rate in the control group (red dashed line) remained at 3.5% ± 0.8%. (Two‐way ANOVA followed by Sidak's multiple comparisons; *n* = 5 animals per group.) (G) Quantification showing elevated number of NeuN/GFP double‐positive cells in 2R 30 + 30D group (2 rounds of AAV‐TFs administration) than that in 1R 60D group (1 round of AAV‐TFs administration) (Unpaired *t*‐test; *n* = 5 animals per group). (H) Typical images showing the co‐labeling of NEUROD1 + DLX2‐infected cells (green) with markers for striatal neurons (DARPP32, CTIP2) (red). Scale bars = 20 μm, 10 μm. (I) Statistics showing the proportion of a series of neuron subtypes in NEUROD1 + DLX2‐infected cells. (*n* = 4 animals per group) (J) Representative images cells for whole cell recordings. Scale bar = 20 μm. (K–M) Typical traces of repetitive action potentials (AP) (K), sodium/potassium current (L) and spontaneous postsynaptic current (sPSC) (M) in GFP‐infected astrocytes in the control group (top panel) and converted neurons (bottom panel) in the ischemic injured striatum. Red asterisk indicates amplified typical trace of sPSC. Note that the *N* + D converted neurons has robust AP, sodium/potassium current, and sPSC. (*N*–R) The statistical graphs comparing the frequency of AP and sPSC (N and Q), as well as the amplitude of AP (O), sodium/potassium current (P) and sPSC (R) among the sham group, GFP group and *N* + D group. Note that the converted neurons share the similar electrophysiological feature to the local neurons in the sham group (One‐way ANOVA followed by Tukey's post hoc test; *n* = 16–18 cells from 3 animals per group).

Next, we explored the fate of the astrocytes expressing the TFs (Figure 3A,B, Figure S2A–B). In control groups, astrocytes infected with the control virus (GFP alone) remained GFAP^+^ astrocytes regardless of one round or two rounds of AAV infection (Figure 3C,E,F, Figure S2C,E,F), indicating that our AAV system is specific to astrocytes in both the cortex and striatum. In contrast, the cells infected with TFs were initially GFAP^+^ astrocytes at the early stage of 10 days post infection but gradually changed their cell fates into NEUN^+^ neurons (Figure 3D, Figure S2D). Quantitative analyses revealed that accompanying a decrease of GFAP^+^ astrocytes among TF‐infected cells (Figure 3E, Figure S2E, blue line), there was an increase of NEUN^+^ neurons among the TF‐infected cells (Figure 3F, Figure S2F, blue line), suggesting a clear cell fate change from astrocytes into neurons. Moreover, the number of converted neurons increased after two rounds of TF‐infection compared to one round of TF‐infection (Figure 3G, Figure S2G), suggesting that two rounds of TF‐infection induced more neuronal conversion than one round of treatment.

We then investigated what kind of neurons were generated after conversion. We found that the majority of converted neurons in the cortex expressed cortical pyramidal neuron markers TBR1, CUX1, and SATB2, with a small portion expressing interneuron markers including PV, NOS, SST, and CALBINDIN (Figure S2H,I, Figure S3A). In the striatum, over half of the converted neurons expressed the striatal GABAergic projection neuron markers DARPP32 and CTIP2, with a small portion expressing interneuron markers such as PV, NOS, SST, and CALBINDIN (Figure 3H,I, Figure S3B). These results suggest that in vivo TF‐mediated AtN conversion can generate appropriate neurons close to the local neuronal identity in the ischemic cortex and striatum when administered at 30 days after stroke.

In order to examine whether the converted neurons were functional and integrated into the local circuit, electrophysiological analyses were performed, focusing on the converted neurons after two rounds of infection. The converted neurons in both ischemic injured cortex and striatum showed large sodium currents, fired repetitive action potentials, and displayed spontaneous postsynaptic events, which were in sharp contrast to the cells infected by control virus (Figure 3J–R, Figure S2J–R). Together, these data indicated that in vivo TF‐based gene therapy generated functional new neurons in the ischemic cortex and striatum that integrated into neural circuits through functional synaptic connections.

### 3.3 Tissue Repair Through AtN Conversion

**FIGURE 4:**
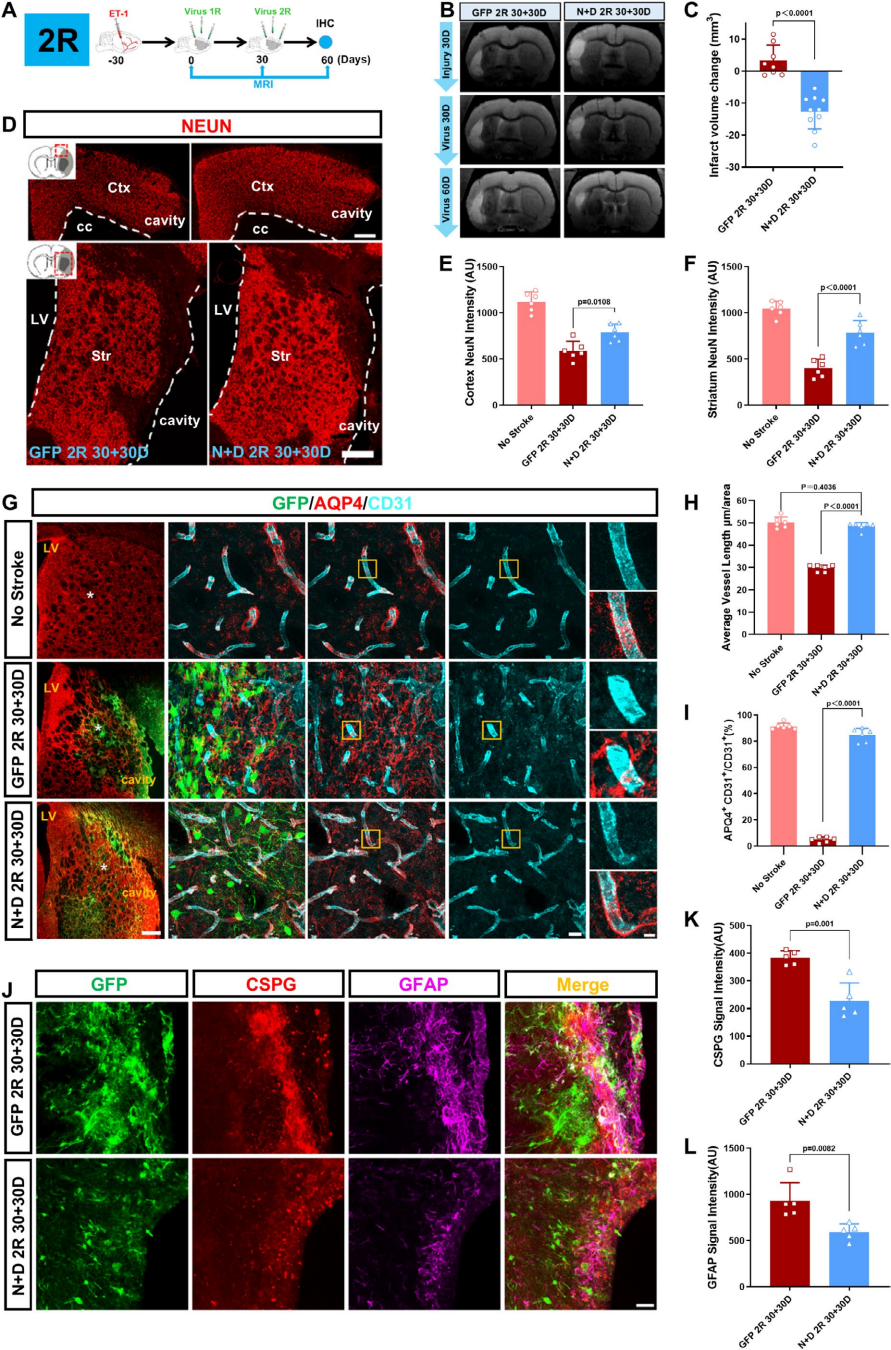
Tissue repair through AtN conversion. (A) Schematic showing the flows of experimental design. (B) T2‐weighted (T2W) images showing the changes of the size of the infarction before (Injury 30D) and after the virus injection (virus 30D and virus 60D). The white highlighted areas suggest the infarct regions. (C) The statistical graph showing the changes of the infarct size after the virus injection (Calculation method: Infarct volume of Virus 60D—Infarct volume of Injury 30D). (One‐way ANOVA followed by Tukey's post hoc test; *n* ≥ 7 animals per group). (D) Representative immunofluorescence staining revealing the neuronal density (indicated by NEUN, red) in the cerebral cortex and striatum of the control group and 2‐round TFs‐treatment group. Note higher density of NEUN^+^ cells in the injured cortex and striatum in the TFs‐treatment group than that in the control group. Scale bar = 500 μm. (E, F) The statistical graph comparing the fluorescence intensity of the neuronal marker NEUN in the cerebral cortex (E) and striatum (F). (One‐way ANOVA followed by Tukey's post hoc test; *n* = 6 animals per group). (G) Typical images showing the distribution of AQP4 (red) in the striatum of no stroke group (top row), the control group (middle row) and the *N* + D 2R group (bottom row). Note that the distribution of AQP4 under normal condition (top row) is to wrap the blood vessels (labeled by CD31, cyan). In the control group (middle row), the AQP4 lost their polarity and mislocated away from the blood vessel, whereas such mislocation of AQP4 partly recovered in the *N* + D 2R group (bottom row). Enlarged views were taken from the position marked by white asterisks. The yellow insets reveal the high magnifications of the relation of AQP4 and blood vessel (labeled by CD31). Scale bars = 500, 20, 10 μm (left to right). (H, I) The statistical graphs comparing length of blood vessels (H), as well as the co‐localization of AQP4 signal and CD31 signal (I) among the no stroke, the control group and *N* + D groups (*N* + D1R and *N* + D 2R). (One‐way ANOVA followed by Tukey's post hoc test; *n* = 6 animals per group.) (J) Representative images illustrating the signal of CSPG (red) and GFAP (magenta) along the edge of the cavity in the ischemic injured striatum among the control group and *N* + D groups. Note that intensity of CSPG and GFAP along the cavity was higher in the control group, whereas in the *N* + D groups it decreased. Scale bar = 50 μm. (K, L) Quantification comparing the fluorescence intensities of CSPG (K) and GFAP (L) among the control group and *N* + D groups (*N* + D 1R and *N* + D 2R). (Unpaired *t*‐test; *n* = 5 animals per group).

After demonstrating successful AtN conversion in the cortex and striatum, we further investigated whether this efficient AtN conversion might have any therapeutic effects. Since two rounds of AAV‐TFs generated more neurons in the injured regions, in the following analyses we focused on the therapeutic effects of two‐round TF treatment. We firstly performed consecutive magnetic resonance imaging (MRI, T2W) on each animal before and after virus injection to evaluate the changes in the infarct volume (Figure 4A). The MRI images showed that compared to the GFP control groups, the infarct volume reduced significantly in the TF‐treated groups (Figure 4B,C). The reduction in the infarct volume after TF treatment was further supported by immunostaining of cell nuclei (DAPI, Figure S4A,B), where the control groups showed significant tissue loss but the TF‐treatment groups showed better tissue preservation. Consistent with tissue repair, the neuronal density in the ischemic cortical and striatal regions, indicated by the intensity of NEUN signal, also showed a significant increase after receiving AAV‐TFs (Figure 4D–F). Besides the elevated NEUN signal, we further investigated the recovery of neurovascular units as another important index for the repair of brain tissue following ischemic stroke. In the GFP control group, the average length of blood vessels (indicated by CD31 signal) became short and the capillary basement membrane (indicated by LAMININ signal) was abnormally thickened, when compared with that in normal tissue (Figure 4G,H, Figure S4C). These abnormal vascular structures partially recovered after the AtN conversion (Figure 4G,H, Figure S4C). In addition, the subcellular distribution of Aquaporin‐4 (AQP4) that was dissociated from the blood vessels in the control groups partially recovered after the AtN conversion (Figure 4G,I). Moreover, in the chronic stage of stroke, glial scars always form along the edge of the lesion cores, serving as a double‐edged sword to inhibit exogenous invasion but also simultaneously impede neural regeneration. After the AtN conversion, the signal of GFAP, CSPG, and NG2 significantly decreased along the edge of the lesion core (Figure 4J–L, Figure S4D–E), suggesting a reduction in glial scar formation following TF treatment. Taken together, these results indicated that AtN conversion repaired neural tissue after stroke by increasing neuronal density, promoting the recovery of neurovascular units, and reducing glial scar formation.

### 3.4 Functional Recovery Through Two Rounds of AtN Conversion

**FIGURE 5:**
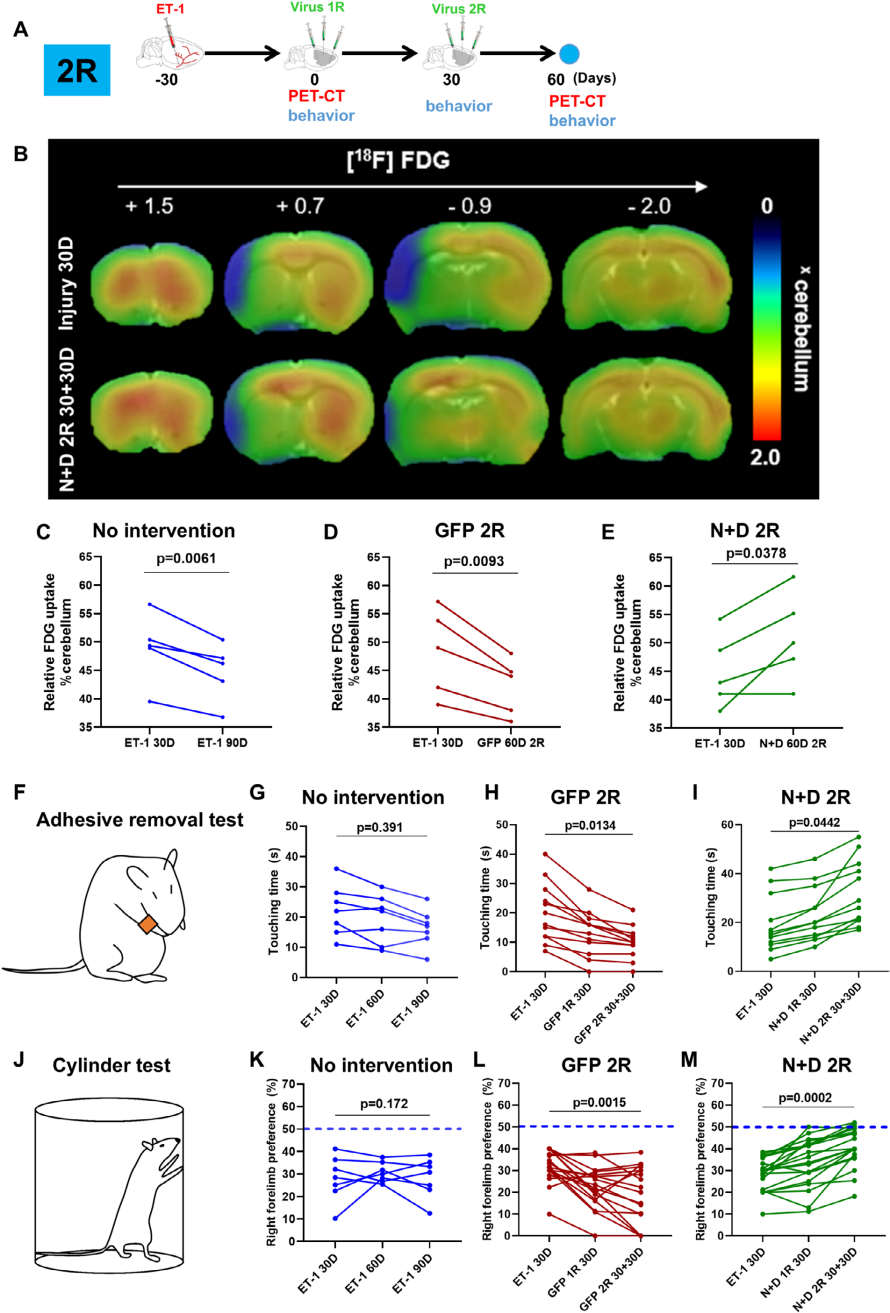
Functional recovery through two‐round AtN conversion. (A) Schematic diagram showing the experimental design. [^18^F] FDG‐PET/CT scans were performed before (30 days after ET‐1 injection) and after the virus injections (30 + 30 days after 2‐round injection). Behavioral studies were conducted before (30 days after ET‐1 injection) and after the virus injections (30 days after 1‐round injection, and 30 + 30 days after 2‐round injection). (B) Representative serial [^18^F] FDG‐PET/CT scans on the coronal plane of brain from anterior (+1.5) to posterior (− 2.0) from the same animal before and after the virus injections. Blue indicates the weak signal, whereas red indicates the strong signal. Note that the size of blue regions on the left side before the virus injection (Injury 30D, top row) declined after 2‐round *N* + D administration (*N* + D 2R 30 + 30D, bottom row). (C–E) The line chart showing the FDG uptake by neurons in the no intervention, the control group, and *N* + D 2R group before and after virus injection. Note that in the no intervention (blue line) and the control group (red line), the signal of FDR continued to decline over time, whereas in *N* + D group (green line) it increased after the treatment. Each line represents an animal conducting the test before and after the virus injection (Paired *t*‐test; *n* = 5 animals for each group). (F) Cartoon diagram of adhesive removal test. (G–I) Animals in the no intervention (G, blue line) and control groups (H, red line) spent decreasing time to touch the stickers over time, whereas animals in *N* + D group (I, green line) spent increasing time to touch the stickers after the treatment (H) (One‐way ANOVA followed by Tukey's post hoc test; *n* = 7 animals in no intervention group; *n* ≥ 11 animals in GFP and *N* + D group; each line represents an animal conducting the test before and after the virus injection). (J) Cartoon diagram of Cylinder test. (K–M) The usage preferences of contralateral forelimb of animals in the no intervention (K) and control group (L) continued to decrease over time, whereas the rate increased to 50% after *N* + D treatment (M) (One‐way ANOVA followed by Tukey's post hoc test; *n* = 7 animals in no intervention group; *n* ≥ 11 animals in GFP and *N* + D group; each line represents an animal conducting the test before and after the virus injection).

After observing tissue repair following AtN conversion, we examined whether AtN conversion might help functional recovery of the brain. Firstly, we performed positron emission tomography (PET) with 2‐deoxy‐2‐[fluorine‐18] fluoro‐D‐glucose (^18^F‐FDG, an analogue of glucose) to evaluate the level of glucose consumption, which corresponds to neuronal activity [38] (Figure 5A). Thirty days after the ischemic injury, the injured regions displayed reduced [^18^F] FDG signal due to neuronal loss (Figure 5B, blue color), and without treatment (the GFP control group and no intervention group), the signal of ^18^F‐FDG continued to decrease over time (Figure 5C,D). In contrast, after two rounds of AtN conversion, the signal of ^18^F‐FDG in the lesion areas increased significantly, suggesting elevated neuronal activity following TF treatment (Figure 5B–E).

**FIGURE 6:**
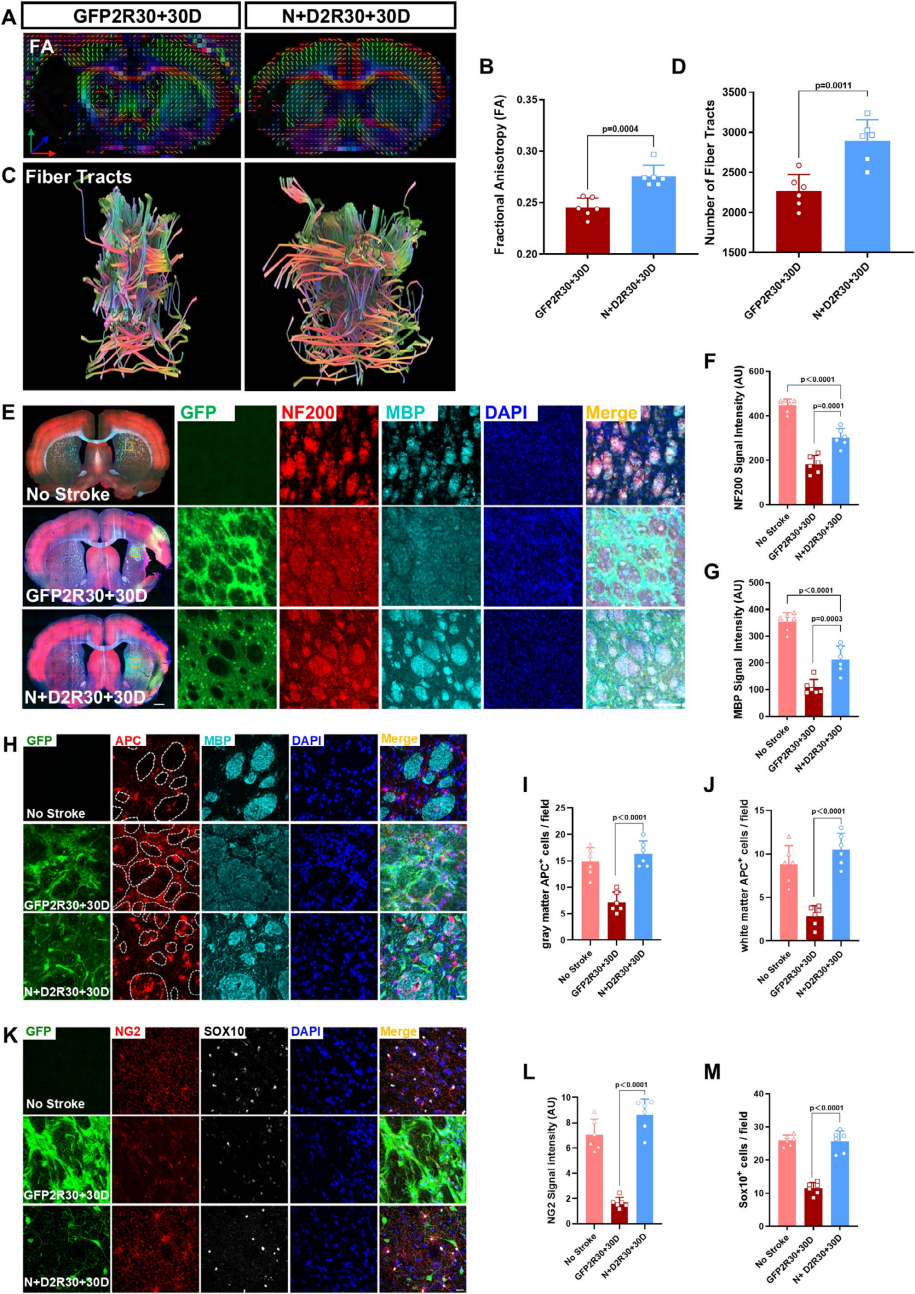
The alleviation of white matter injury after treatment. (A) Reconstructed images of the directional preference of diffusion [fractional anisotropy (FA) based on MRI‐DTI sequence scanning. (B) Quantification of the FA counted using Dsi‐studio (Unpaired *t*‐test; *n* = 6 animals per group). (C) Reconstructed images illustrating fiber bundles passing through the damaged area based on MRI‐DTI sequence scanning. (D) Quantification of the number of fiber bundles passing through the damaged area using Dsi‐studio (Unpaired *t*‐test; *n* = 6 animals per group). (E) Representative images showing the expression of NF200 (red) and MBP (cyan) in control group (top raw) and *N* + D 2R group (bottom raw). The insets are enlarged views of virus‐infected area (GFP^+^, green) showing the expression of NF200 and MBP. Note the increased signal of NF200 and MBP *N* + D 2R group. Nuclei were counterstained with DAPI (blue). Scale bars = 1000 μm, 50 μm. (F–G) The statistical analysis showing increased fluorescence intensity of NF200 (F) and MBP (G) in *N* + D groups. (One‐way ANOVA followed by Tukey's post hoc test; *n* = 6 animals per group.) (H) Images showing that *N* + D 2R group had more APC^+^ cells (red) in the injured striatal axon buddle (labeled by MBP, cyan) than the control group. White dashed circles delineate striatal axon buddle. Nuclei were counterstained with DAPI (blue). Scale bar = 20 μm. (I–J) The statistical graph revealing the increased number of APC‐positive cells in both the gray matter (I) and white matter (J) of the *N* + D groups (One‐way ANOVA followed by Tukey's post hoc test; *n* = 6 animals per group). (K) Representative images showing more NG2^+^ (red) and SOX10^+^ (white) cells in the striatum in *N* + D groups. Nuclei were counterstained with DAPI (blue). Scale bar = 20 μm. (L–M) Quantification illustrating the increasing fluorescence intensity of NG2 and the increasing density of SOX10‐positive cells in *N* + D groups (One‐way ANOVA followed by Tukey's post hoc test; *n* = 6 animals per group).

Next, we performed behavioral tests to further evaluate whether AtN conversion could bring functional recovery (Figure 5A). Since the cortical damages in this model were located mainly in the somatosensory cortex, we first used the adhesive removal test to examine the sensory ability of each animal with or without the TF‐treatment (Figure 5F). The results showed that the animals in the GFP control group or no intervention group showed a continuous decrease or no improvement in the time of touching the tape wrapping around the forelimb, respectively, indicating an impairment in the sensory ability (Figure 5G,H). In contrast, the animals receiving the TF‐treatment showed an increase in the time of touching the tape, suggesting a recovery of sensory ability after the treatments (Figure 5I). Besides the sensory ability, we also performed the cylinder test to evaluate the right forelimb motor ability after left side ischemic injury (Figure 5J). The results showed that the utilization rate of the right forelimb continued to decline or remained at a low level in the GFP control group or no intervention group, respectively (Figure 6K,L); whereas after TF‐treatment, the utilization rate of the injured forelimb increased continuously after two rounds of AtN conversion (Figure 5M), suggesting the recovery of motor ability after the treatments. In summary, these results suggest that the two rounds of AtN conversion promote functional recovery after stroke.

### 3.5 Alleviation of White Matter Injury After AtN Conversion

Previous studies mainly focused on the repair in the gray matter where AtN conversion takes place. However, white matter injury is also significant after ischemic stroke [39, 40], raising an important question of whether AtN conversion can rescue white matter injury after stroke. To answer this question, we first used diffusion tensor imaging (DTI) to examine the change of white matter after the AtN conversion. DTI is a magnetic resonance imaging modality evaluating the orientation and integrity of white matter (WM) [41, 42]. After reconstructing the WM tracts based on DTI imaging, we found that compared with the control group (GFP 2R30 + 30D), both the fractional anisotropy (FA) and the number of nerve fibers (NF) passing through the injured regions increased in the TF‐treated group (*N* + D 2R30 + 30D) (Figure 6A–D), suggesting an improvement in WM integrity after AtN conversion. The rescue of axonal fibers was further supported by the increased signal of NF200 (a marker of neuronal axons) within the striatal axon bundles and corpus callosum after AtN conversion (Figure 6E,F, Figure S5A,B). Within the axonal bundles, we observed some GFP positive fibers expressing NF200 in the corpus callosum (Figure S5A), suggesting that the newly converted neurons in the cortex projected axons to their synaptic targets through these existing axon bundles. In addition to the axonal fibers, axonal myelination indicated by MBP signal was also partially restored after AtN conversion (Figure 6E,G, Figure S5A,B). Meanwhile, the number of mature oligodendrocytes (indicated by APC^+^) and oligodendrocyte precursor cells (indicated by NG2^+^SOX10^+^) also increased after the TF treatment (Figure 6H–M). To determine possible reasons for the increased myelination, we counted the number of oligodendrocytes at different time points after the virus injections. The quantities of oligodendrocytes (indicated by SOX10) were comparable between the control and treatment groups at the early stage of AtN conversion (10 DPI, Figure S5C,D). At later stages, the number of oligodendrocytes gradually increased in the TF group, whereas in the control group it remained at a relatively low level (Figure S5C,D). These results suggest that AtN conversion may promote re‐myelination in the ischemic areas through enhancing the proliferation of oligodendrocytes. Together, our studies suggest that the TF‐mediated AtN conversion may alleviate the white matter injury through increasing axonal fibers and promoting axonal myelination after ischemic injury.

### 3.6 Transcriptome‐Wide Recovery Following AtN Conversion in the Ischemic Striatum

**FIGURE 7:**
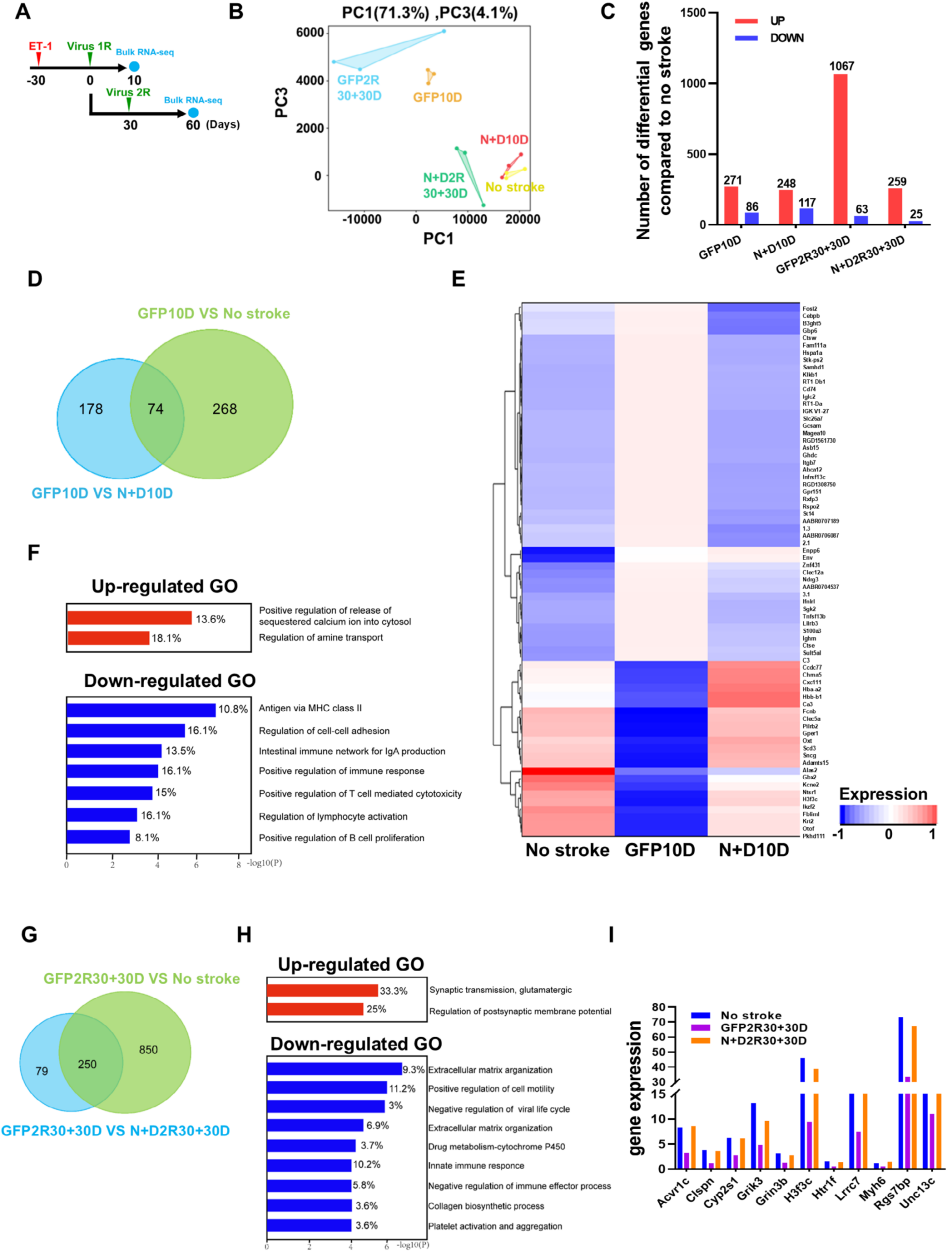
Transcriptome‐wide recovery following NEUROD1 + DLX2‐mediated AtN conversion in the ischemic striatum. (A) The schematic shows experiment design. Striatal tissues were collected from no stroke health rats (No stroke), ET‐1 induced MCAO‐like rats receiving 1st round of control and TFs viruses at 10 D (GFP10D, *N* + D10D), and ET‐1 induced MCAO‐like rats receiving 2nd round of control and TFs viruses at 30 + 30 D (GFP2R30 + 30D, *N* + D2R30 + 30D). *n* = 3 animals for each group. (B) Principal component analysis (PCA) of all samples based on gene expression profile. (C) Upregulated (red column) and down‐regulated (blue column) differential expressed genes (DEGs) from the comparison of GFP10DPI, GFP2R30 + 30D and *N* + D2R30 + 30D with NO Stroke, respectively. DEGs are defined as at least 2‐fold change among samples, and *p* value < 0.01. (D) Venn diagram shows the comparison of differentially expressed genes (DEGs) between GFP10D group and no stroke group with those between GFP10D and *N* + D10D. Note that there was shared 74 DEGs. (E) Hierarchical clustering and heat‐map of the overlapped 74 DEGs based on (D). Red indicates high read count levels, whereas blue indicates low read count level. Note the similarity of heatmap pattern between *N* + D10D group and no stroke group. (F) GO enrichment of the overlapped 74 DEGs based on (D). Red illustrates upregulated GO terms, whereas blue illustrates the down‐regulated GO terms. (G) The Venn diagram showing the comparison of DEGs between GFP2R30 + 30D and no stroke with those between *N* + D2R30 + 30D versus GFP2R30 + 30D. Note that there were shared 250 DEGs. (H) GO enrichment of the overlapped 250 DEGs based on (G). Red illustrates upregulated DEGs in the *N* + D2R30 + 30D group compared with GFP2R30 + 30D group, whereas blue illustrates the down‐regulated DEGs in the *N* + D30 + 30D group compared with GFP30 + 30D group. (I) Histogram comparing the expression level of 11 upregulated shared DEGs based on (G) among No stroke, GFP2R30 + 30D and *N* + D2R30 + 30D group.

We previously used bulk RNA sequencing to investigate the global changes in the ischemic cortical regions after NEUROD1‐mediated AtN conversion and found that NEUROD1 upregulated genes related to neurogenesis when it was transduced to reactive astrocytes in the subacute phase [19]. In this study, we generated MCAO‐like stroke that induced much wider injury areas including the striatum and conducted the AtN conversion studies in the chronic stage of the ischemic injury. To better understand the global changes in ischemic striatum after NEUROD1 + DLX2‐mediated AtN conversion and investigate the possible repairing mechanisms, we conducted RNA sequencing (RNA‐seq) analysis to compare the transcriptome profiles between the GFP control and TF groups at short‐term and long‐term after viral injection (Figure 7A). Based on the principal‐component analysis (PCA) of the overall genome‐wide expression, the relationship between NEUROD1 + DLX2‐infected tissues after stroke and healthy striatal tissues (no stroke) was much closer than that infected by the GFP control virus at both early and later sampling times after stroke (Figure 7B). Differentially expressed genes (DEGs, using fold change > 2.0, *p* value < 0.05) revealed a huge difference in gene‐expression profiles between no stroke healthy tissues and control virus‐infected stroke tissues at 60 DPI, with a total of 1130 DEGs identified (Figure 7C). In contrast, only 284 DEGs were identified between TF‐infected stroke tissues and no stroke healthy tissues at 60 DPI (Figure 7C). At 10 DPI, the numbers of DEGs in injured tissues from control and NEUROD1 + DLX2 groups were comparable (357 DEGs in GFP‐10 DPI and 365 DEGs in *N* + D‐10 DPI, Figure 7C), suggesting that dramatic improvement took place at the late stage of AtN conversion. To figure out how AtN conversion promotes the repair of the ischemic tissue, we compared the DEGs between NEUROD1 + DLX2‐infected and control AAV‐infected tissues with those between healthy and control AAV‐infected tissues. The Venn diagram showed that at 10 DPI there were 74 shared DEGs (Figure 7D). The expression pattern of these 74 DEGs in NEUROD1 + DLX2‐infected tissues was similar to that in healthy tissues but distinct from that in the control virus‐infected tissues (Figure 7E). GO term analysis indicated that the downregulated DEGs were mainly related to immune response (e.g., Antigen via MHC class II, intestinal immune network for IgA production, and positive regulation of T cell mediated cytotoxicity), whereas the upregulated DEGs might be related to synaptic functions (positive regulation of release of sequestered calcium ion into cytosol and regulation of amine transport, Figure 7F). At a later stage (30 + 30D), the number of shared DEGs between the GFP control stroke tissues versus no stroke healthy tissues and the GFP control stroke tissues versus TF‐treated tissues increased to 250 (Figure 7G), and the majority of them were downregulated (239 DEGs down‐regulated vs. 11 DEGs upregulated, Figure 7H). GO term analysis showed that the top downregulated DEGs were related to glial scar formation (e.g., extracellular matrix organization, positive regulation of cell motility, collagen metabolic process and collagen biosynthetic process) and immune response (e.g., negative regulation of viral life cycle, innate immune response) (Figure 7H). In addition, pathways involved in cytochrome P450 metabolism (Drug metabolism‐cytochrome P450) and platelet degranulation were also downregulated in the NEUROD1 + DLX2‐infected striatal tissues at 60 DPI. The blockage of cytochrome P450‐20‐hydroxyeicosatetraenoic acid (20‐HETE) has been reported to reduce infarct volume and improve the neurological outcome in rat and monkey stroke models [43, 44]. Platelet activation increases in the acute phase of the stroke and gradually decreases to the normal level in the recovery stage, suggesting the reconstitution of blood–brain barriers. Downregulation of these two pathways is consistent with the above observations of reduced damage areas and restored neurovascular units. Within the 11 upregulated DEGs, six genes encode proteins participating in synaptic functions (*Grik3, Grin3b, Htr1f, Lrrc7, Rgs7bp, Unc13c*), and two of them encode proteins involving neuronal survival (*Acvr1c*) and active transcription (*H3f3c*, Figure 7I). GO term analysis implied that the upregulated DEGs in the late stage were also associated with synaptic functions (synaptic transmission and regulation of postsynaptic membrane potential), and that the matching score was higher than that in the early stage (higher enrichment scores, 33.3%/25% from *N* + D2R30 + 30D versus13.6%/18.1% from *N* + D10D, Figure 7F,H). Together, these data suggest that in the early stage, ectopic expression of NEUROD1 + DLX2 in the reactive astrocytes may reduce inflammation, and in the late stage, AtN conversion may promote synaptic functions and alleviate glial scar formation.

## 4 Discussion

In this study, we demonstrate that in vivo astrocyte‐to‐neuron conversion mediated through transcription factor‐gene therapy can efficiently regenerate a large number of functional new neurons in the cortex and striatum in a chronic ischemic stroke model and achieve functional rescue of both sensory and motor deficits in rodent animals. The converted neurons intermingled with the local neurons and prevented tissue loss in the injury sites. The deterioration of blood vessels and formation of glial scar in the ischemic region were ameliorated. In addition, we observed more myelinated axon bundles in the ischemic white matter after in vivo astrocyte‐to‐neuron conversion. RNA‐seq revealed substantial attenuation of inflammation and synaptic recovery after the transcription factor‐mediated astrocyte‐to‐neuron conversion. All this tissue repair led to the evident improvement of the sensory and motor behavioral deficits. This study expands the application scope of in vivo AtN conversion from the previous restricted brain region to multiple brain regions and suggests a broad therapeutic time window of in vivo AtN conversion to treat ischemic stroke.

### 4.1 A Broad Intervention Time Window of in Vivo Astrocyte‐To‐Neuron Conversion

One major challenge of many therapeutic interventions of ischemic stroke is the narrow time window. Current clinical treatment for stroke is restricted to recanalization and restoration of cerebral blood flow, including both intravenous thrombolysis and mechanical thrombectomy, but only a limited number of patients are eligible for these time‐sensitive treatments [45]. Other strategies, including neuroprotective approaches and rehabilitation therapies, display magnificent therapeutic effects within hours, days, or weeks after the onset of stroke [46]. Therefore, developing the strategies for the patients in the late subacute and the chronic phase of stroke is demanding. Cell transplantation therapy in the preclinical studies has a relatively broad intervention time window in the stroke models from days to months after the insult [47, 48]. But cell transplantation encounters the challenges of batch variation and tumorigenesis [48, 49]. In vivo astrocyte‐to‐neuron conversion is a new promising approach to use the endogenous astrocytes to regenerate neurons. Previous studies selected acute or early subacute rodent stroke models to conduct NEUROD1‐mediated in vivo AtN conversion and demonstrate that it can promote both the tissue repair and functional recovery [19, 24, 25, 26, 50, 51]. Another pilot study using the non‐primate ischemic stroke models has proven that NEUROD1 is able to convert reactive astrocytes to neurons from 10 to 30 days following the onset, suggesting that in vivo AtN conversion may have a broad therapeutic time window [18]. This study provided a series of evidences in the rodent ischemic stroke model to support the hypothesis that transcription factor‐mediated AtN conversion can repair both the tissue and functional deficits in the chronic phase of the disease, broadening its intervention time window.

### 4.2 Multiple Rounds of AAV‐TF Administration Enhance the Numbers of Neurons

Another challenge to treat stroke, especially that passes the acute treatment, is how to regenerate/preserve more neurons in the ischemic region. Previous studies show that endogenous neural stem cells and astrocytes are able to generate neurons after stroke [52, 53, 54]. DCX positive immature neurons/neuroblasts differentiated from neural stem cells in both subventricular zone of the lateral ventricle or subgranular zone of the hippocampus can migrate into the ischemic region [55, 56]. Reactive astrocytes adjacent to the lesion core can dedifferentiate into neuroblasts through upregulating the expression of the neural transcription factor Ascl1, which is stimulated by the WNT2 signal from the dying neurons, exosomes secreted from the blood vessel endothelial cells, or the VEGF signal [57, 58, 59]. However, all these beneficial effects coming from the endogenous cells are short‐lived, which take place within a week after the onset of stroke. The number of DCX positive cells dramatically decreases afterwards, and the number of mature neurons is quite limited. Ectopic expression of neural transcription factors in the astrocytes augments their latent potency to generate neurons, but the number of regenerated neurons depends largely on the delivery systems. Studies using retrovirus or lentivirus to deliver transcription factors only generated quite a small number of neurons; hence, the reduction of the injured areas was not evident [25, 60]. AAV is an efficient viral system that has a broad infection area [61]. Therefore, in the previous studies to treat mouse cortical ischemic injuries, one injection of AAV‐NEUROD1 is sufficient to achieve tissue repair and functional recovery [19]. Because in the clinic the patients with unsatisfactory prognosis also have large infarct areas, we establish a rat MCAO‐like ischemic stroke model with large ischemic regions including the cortex and striatum to mimic these. Considering the extremely large injured area in the chronic stage, we tried multiple rounds of AAV‐TF injection. The second round of AAV‐TF administration increases the pool of start cells through infecting the reactive astrocytes that are not transduced in the first round and hence regenerates more neurons than the single dose of AAV‐TF administration. Importantly, it leads to an increased number of regenerated neurons in the injured sites. This study suggests that in vivo astrocyte‐to‐neuron conversion mediated through transcription factor‐gene therapy could be redosed, especially for the ischemic injury with a large scale. This is fundamentally important to the future clinical translation.

### 4.3 Repair Mechanism More Than Neuronal Regeneration

Beside the regeneration of new neurons to replenish the lost neurons in the injured sites, neuronal protection is another benefit brought by AtN conversion [19]. In this study, we further found the amelioration of the deterioration of the blood vessels, correction of the mislocalization of water channel protein AQP4, as well as the reduction of glial scar formation after AtN conversion. The recovery of blood supply to the ischemic region is critical to the preservation of neural tissue [62]. Incorrect subcellular distribution of AQP4 impairs the glymphatic drainage system to remove the metabolic waste from the brain [63]. Both the recovery of blood vessels and the distribution of AQP4 suggest an improved circulation system in the injured site after AtN conversion. In addition, glial scar impedes the axonal regeneration and the attenuation of glial scar formation after AtN conversion is closely related to the white matter repair and functional recovery of the ischemic animals [64, 65, 66]. Taken together, these results indicate that TF‐mediated AtN conversion has other repair mechanisms in addition to neural regeneration and protection. In order to illuminate the possible repair mechanism, we employed RNA‐seq and found that at the early stage of AtN conversion, ectopic expressing TFs in the reactive astrocytes dramatically reduced the immune and inflammation response in the injured region. Inflammation is a major cause of the second injury after stroke, which leads to further deterioration of the penumbra region [67, 68]. From this study, we observed that even 30 days after the ischemic injury, the second injury in the rat ischemic regions still continued. The reduction of immune and inflammation signals in the early stage of AtN conversion suggests that TFs may first downregulate the pro‐inflammation signal of the reactive astrocytes and then convert them to neurons. As an important part of the inflammation cascade, reactive astrocytes play opposite roles at different stages of stroke [69, 70, 71]. Reactive astrocytes are considered to be protective within 7 days after stroke, but toxic afterwards [11, 71, 72, 73]. A study reported that reactive astrocytes engulfed the synapse of the preserving neurons and led to the functional deficit from the second week in mice ischemic models [74]. The RNA‐seq data shows partial improvement of synaptic functions before the AtN conversion, suggesting that TFs may downregulate the reactive astrocyte to engulf the synapses. At the late stage when most TF‐expressed astrocytes convert to neurons, the synaptic function is further improved due to the new neurons and new connections. In the aspect of the mechanism on neurovascular repair, we think that it may also be related to changes of the reactive astrocytes induced by neural TFs. As an important compartment of the brain–blood barrier (BBB), proliferative astrocytes located at juxtavascular sites in the injured areas participate in vascular repair and the reconstruction of the BBB [73, 75]. It has been reported that in vivo AtN conversion promoted proliferation of local astrocytes [76], which may facilitate the neurovascular repair. In addition, excessive inflammation also deteriorates neurovascular units [77]. Reactive astrocytes aggravate the inflammatory response in many ways, including the production of a large number of inflammatory factors to activate immune cells and increase the BBB leakage [78], spreading the inflammatory signal to adjacent cells through upregulation of gap junction protein (CX43) [79], and impairing the glymphatic drainage to result in the accumulation of damage‐associated molecular patterns that aggravate the inflammation [80, 81, 82]. The role of NeuroD1 to attenuate reactive astrocytes has been reported in other acute injury models [51, 76]. In this study, we find that NEUROD1 + DLX2 can also play the anti‐inflammation role in the chronic injury models, which may be another repair mechanism beside neuronal regeneration to achieve the tissue repair and functional improvement.

### 4.4 Alleviation of the White Matter Damage After AtN Conversion

Given the high incidence of white matter injury after stroke [39], we also explored whether in vivo AtN conversion had any benefits to the white matter repair. In this study, we observed contralateral axonal projections of converted neurons through the corpus callosum, which may replenish the injured axon bundles. In addition, the number of oligodendrocytes gradually increases during the AtN conversion. After ischemic stroke, although oligodendrocyte progenitor cells are able to proliferate and differentiate to promote the white matter repair, such spontaneous white matter repair attenuates at the chronic stage [83, 84, 85]. When the neural TFs were ectopically expressed in the reactive astrocytes in the chronic stage of rat MCAO models, we discovered that the number of oligodendrocytes was augmented after the AtN conversion. Axon growth or regeneration promotes the proliferation of oligodendrocyte progenitor cells, and the oligodendrocytes in the absence of axons are subject to programmed death [86, 87]. Axons projected from the converted neurons and the increased number of axon fibers in the bundles may be the reason for more oligodendrocytes and myelination in the injury sites. Moreover, the continuous inflammation during the prolonged secondary injury after stroke also damages the preserved axons and oligodendrocytes [40]. Therefore, the improvement of the inflammatory microenvironment by ectopically expressing TFs may be another reason for the white matter repair.

### 4.5 Limitation of This Study

This study also has some limitations. Firstly, depending on the viral GFP labeling, rather than stringent approaches including lineage tracing or single‐cell transcriptomic studies [88, 89], we inferred TFs‐mediated in vivo AtN conversion in the chronic phase of the rat MCAO‐like model. The lack of transgenic rats to specifically label the astrocytes and trace the whole process of AtN conversion is the major obstacle. Using retrograde AAVs to pre‐label the pre‐existing neurons may be used to discriminate the converted neurons from the pre‐existing neurons, although the limited labeling rate is the major concern [90]. Secondly, although the anti‐inflammation of ectopically expressing TFs in the reactive astrocytes may be another repair mechanism to both gray matter and white matter, the detailed mechanism by which TFs reduce the global inflammation through reactive astrocytes needs to be further dissected. Thirdly, although 30 days after the stroke is considered the chronic stage in rodent models, the chronic stage of stroke patients is 6 months after the onset, when all pathogenesis and spontaneous recovery events stop [47]. In this rat MCAO‐like model, the neural tissue further deteriorated until 60 days after the stroke, when cavities form and the adjacent GFAP signal decreased compared with that 30 days after the stroke. This pathological state mimics that of stroke patients 6 months after the ischemic insult [47]. Whether in vivo AtN conversion can be applied to treat stroke at a very late stage of the disease is an open question. Future work is required to explore the therapeutic efficacy of in vivo AtN conversion in rodent ischemic models several months or later after the onset of stroke.

## 5 Conclusion

In this proof‐of‐principle study, we provide evidence that multiple rounds of transcription factor‐based gene therapy can efficiently regenerate neurons in a relatively large‐scale ischemic stroke model in the chronic phase, rescue tissue atrophy, reduce scar formation, promote the recovery of neurovascular units, repair the white matter, and enhance functional recovery of the brain.

## Author Contributions

Study conception and design: W.L., G.C., T.W., W. Lei; study implementation: T.W., X.W.; Bioinformative analysis: S.L.; study assistance: M.L., K.W., J.W., K.Z.; MRI and PET image collection: K.Z., L.W., H.X.; data analysis except bioinformative analysis: T.W., X.W.; manuscript drafting: W.L., G.C., T.W., W. Lei. All authors discussed and approved the final version of the manuscript.

## Conflicts of Interest

G.C. is a co‐founder of NeuExcell Therapeutics Inc. The other authors have no conflicts of interest.

## Supporting information


**Figure S1** (related to Figure 2). Multiple rounds of AAV administration enhanced infection areas and number of infected cells.

**Figure S2** (related to Figure 3). Two rounds of AAV‐mediated AtN conversion in cortex.

**Figure S3** (related to Figure 4). The converted neuron acquired the local neuronal subtypes.

**Figure S4** (related to Figure 5). Tissue repaired achieved by AtN conversion.

**Figure S5** (related to Figure 6). White matter repair achieved by AtN conversion.

**Table S1** Primary antibodies.

## Data Availability

The data that support the findings of this study are available from the corresponding authors upon reasonable request.
