# Supplementary figures and images for: Transcription Factor‐Based Gene Therapy Enables Functional Repair of Rat Following Chronic Ischemic Stroke
Source: CNS Neurosci Ther. 2025 May 22;31(5):e70448. doi: 10.1111/cns.70448 (PMC12096174; doi:10.1111/cns.70448)

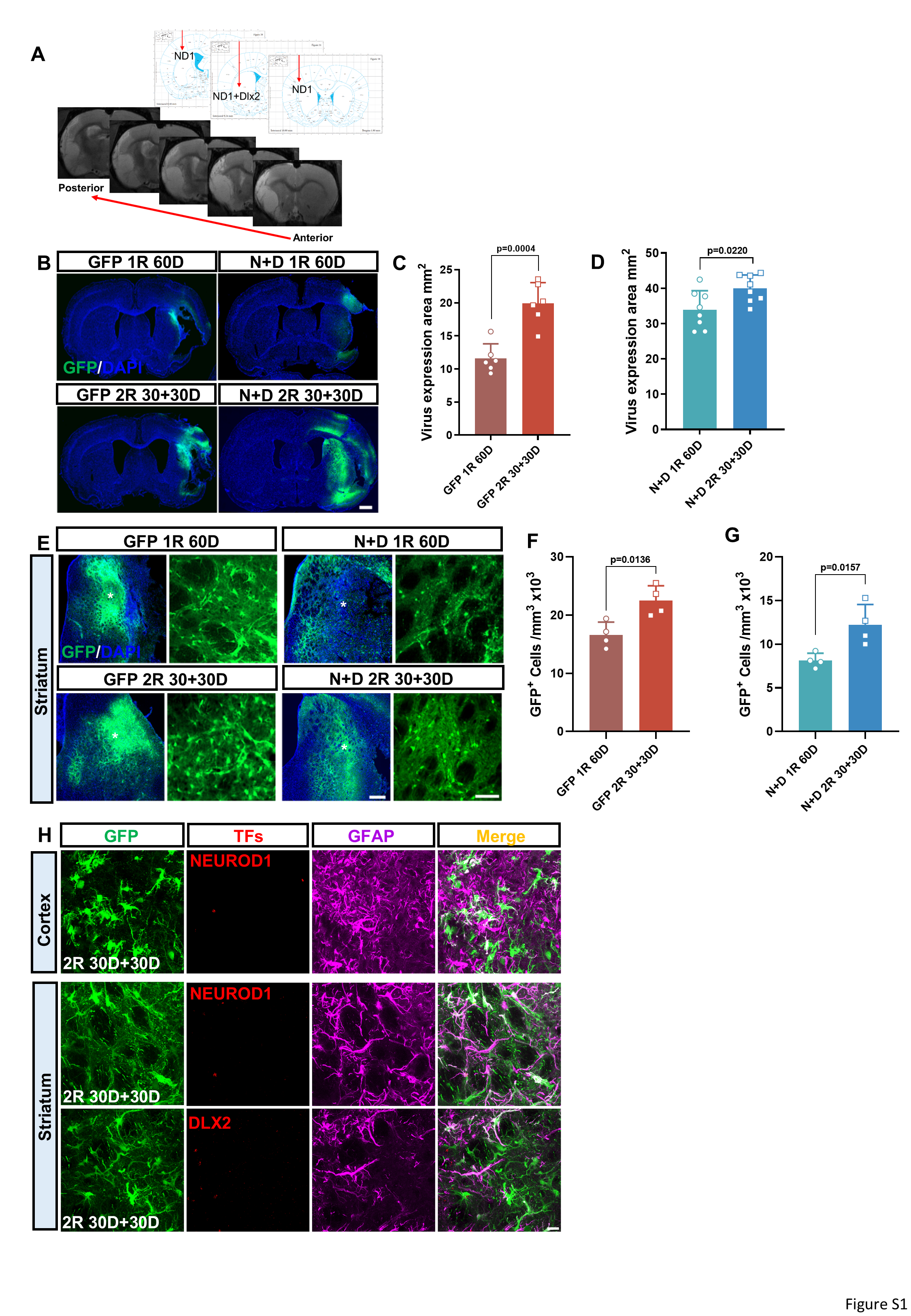

Supplement: Supplementary file 1 — Figure S1 (related to Figure 2). Multiple rounds of AAV administration enhanced infection areas and number of infected cells. [file CNS-31-e70448-s005.tif]

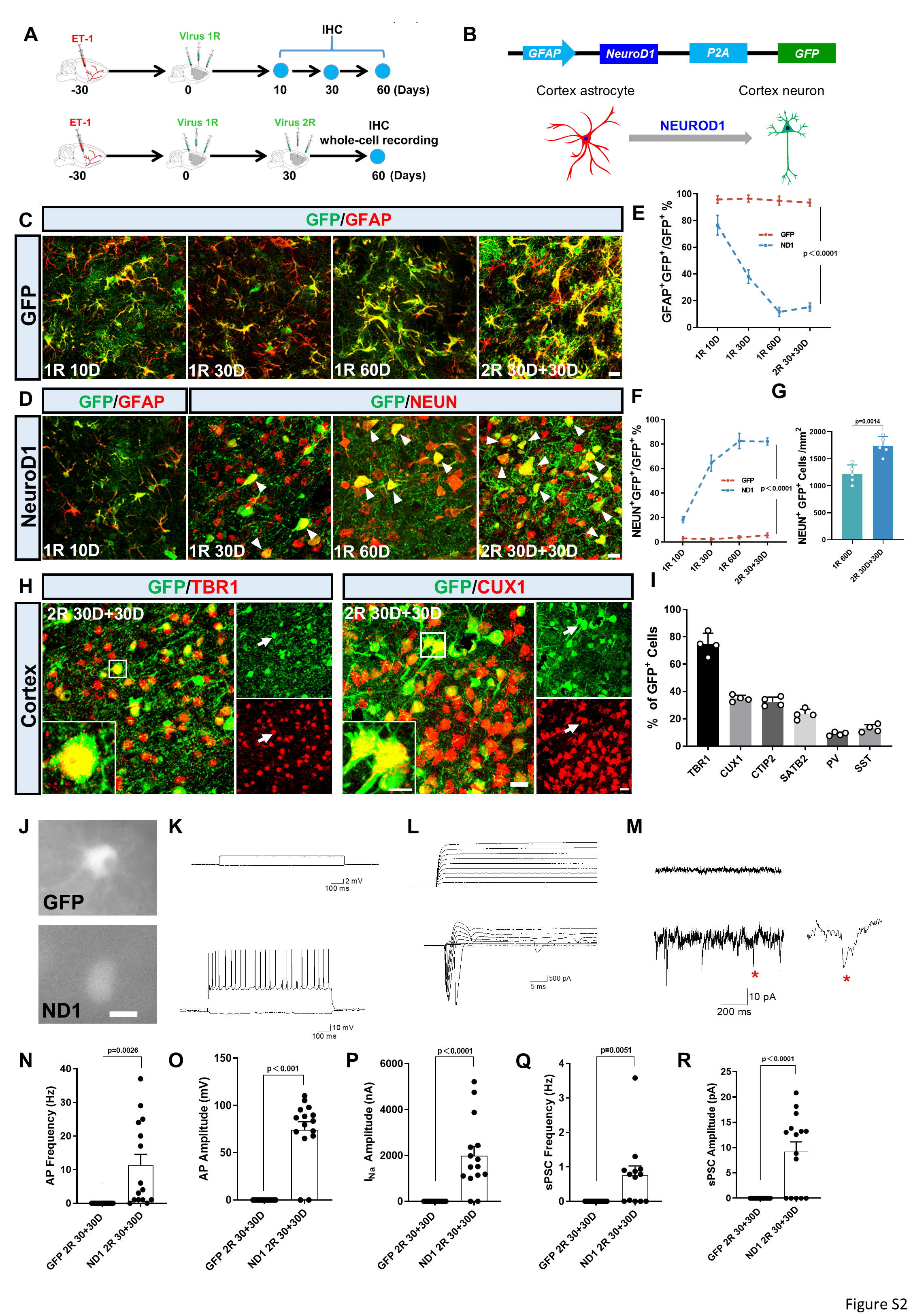

Supplement: Supplementary file 2 — Figure S2 (related to Figure 3). Two rounds of AAV‐mediated AtN conversion in cortex. [file CNS-31-e70448-s001.tif]

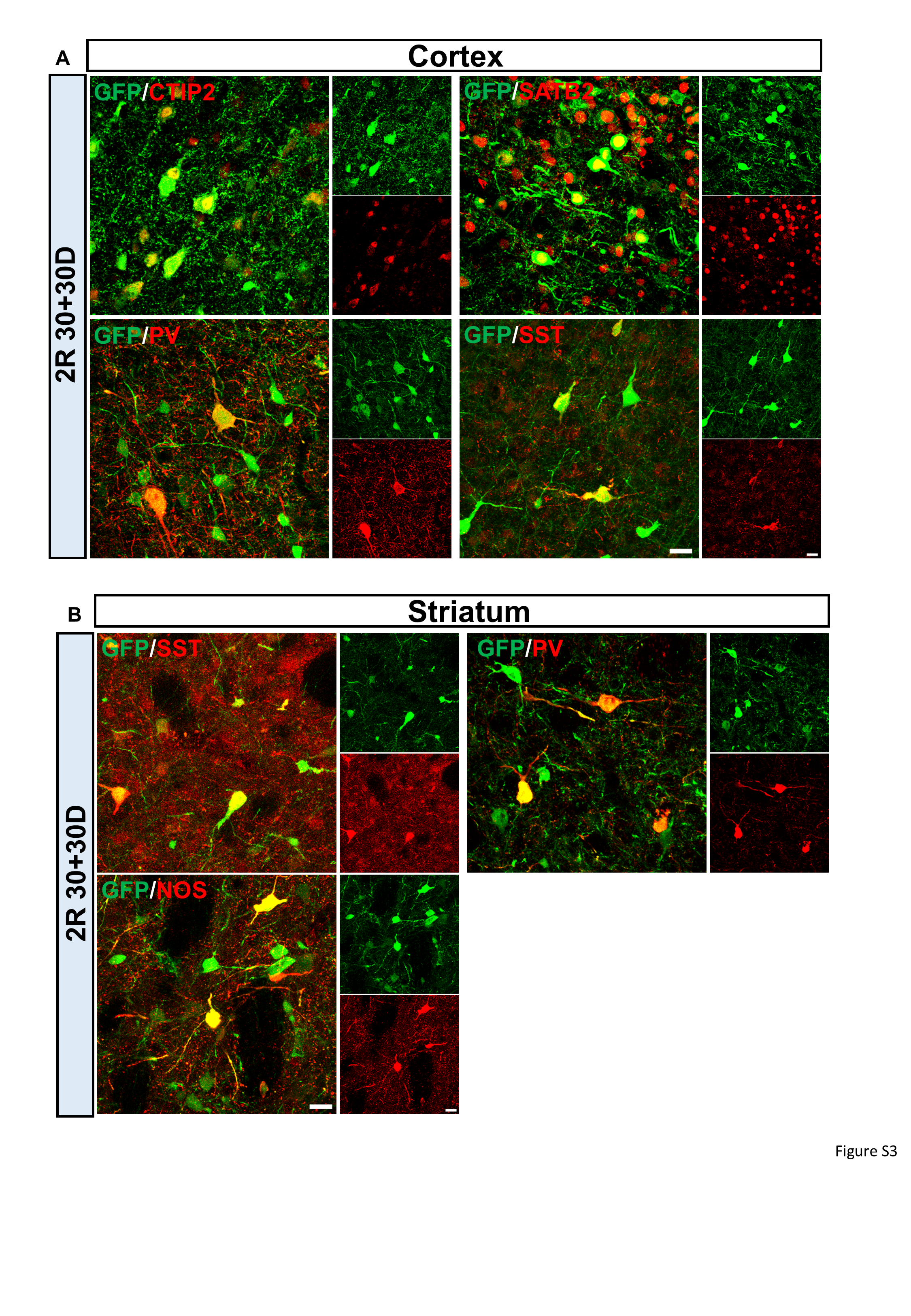

Supplement: Supplementary file 3 — Figure S3 (related to Figure 4). The converted neuron acquired the local neuronal subtypes. [file CNS-31-e70448-s003.tif]

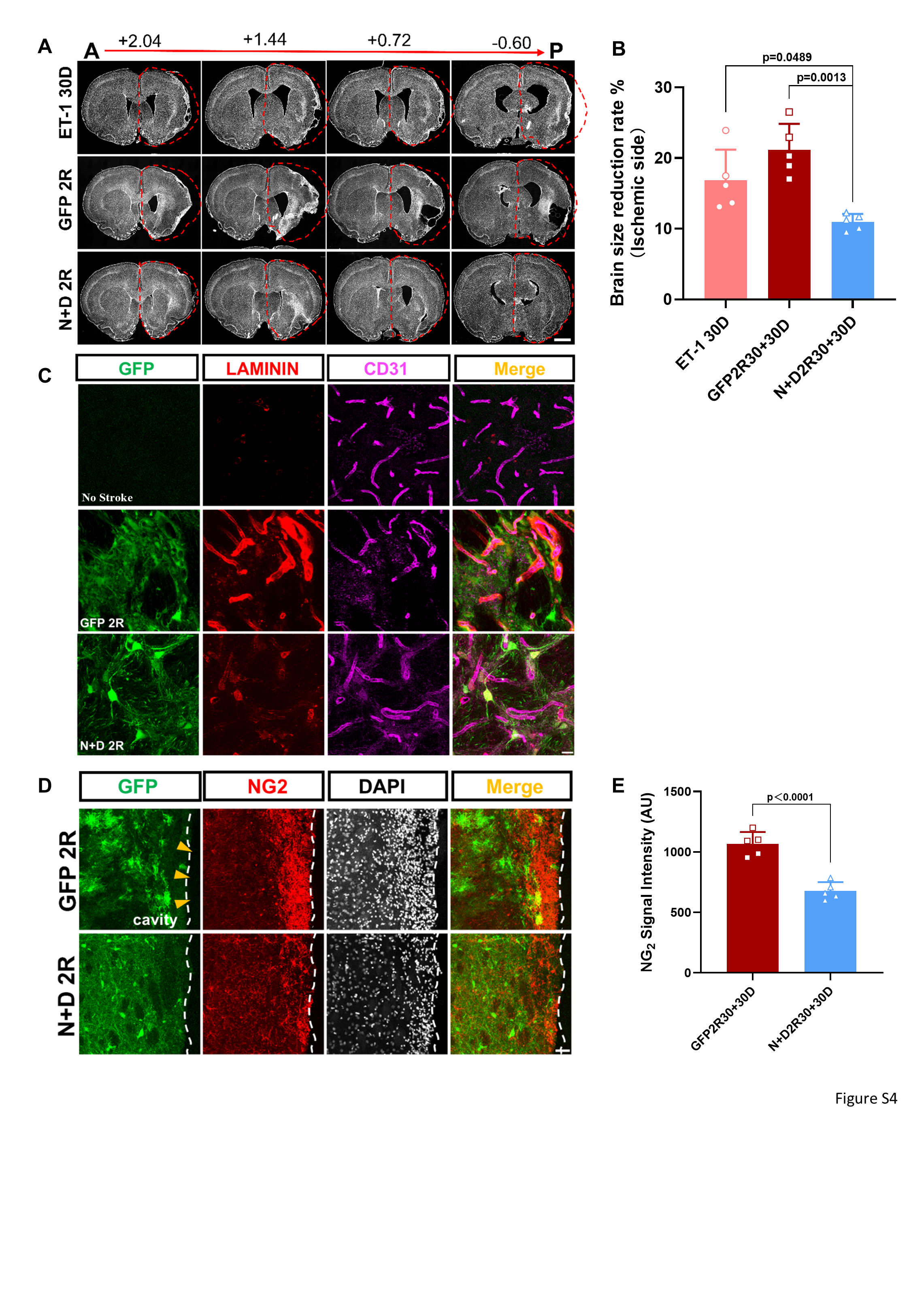

Supplement: Supplementary file 4 — Figure S4 (related to Figure 5). Tissue repaired achieved by AtN conversion. [file CNS-31-e70448-s002.tif]

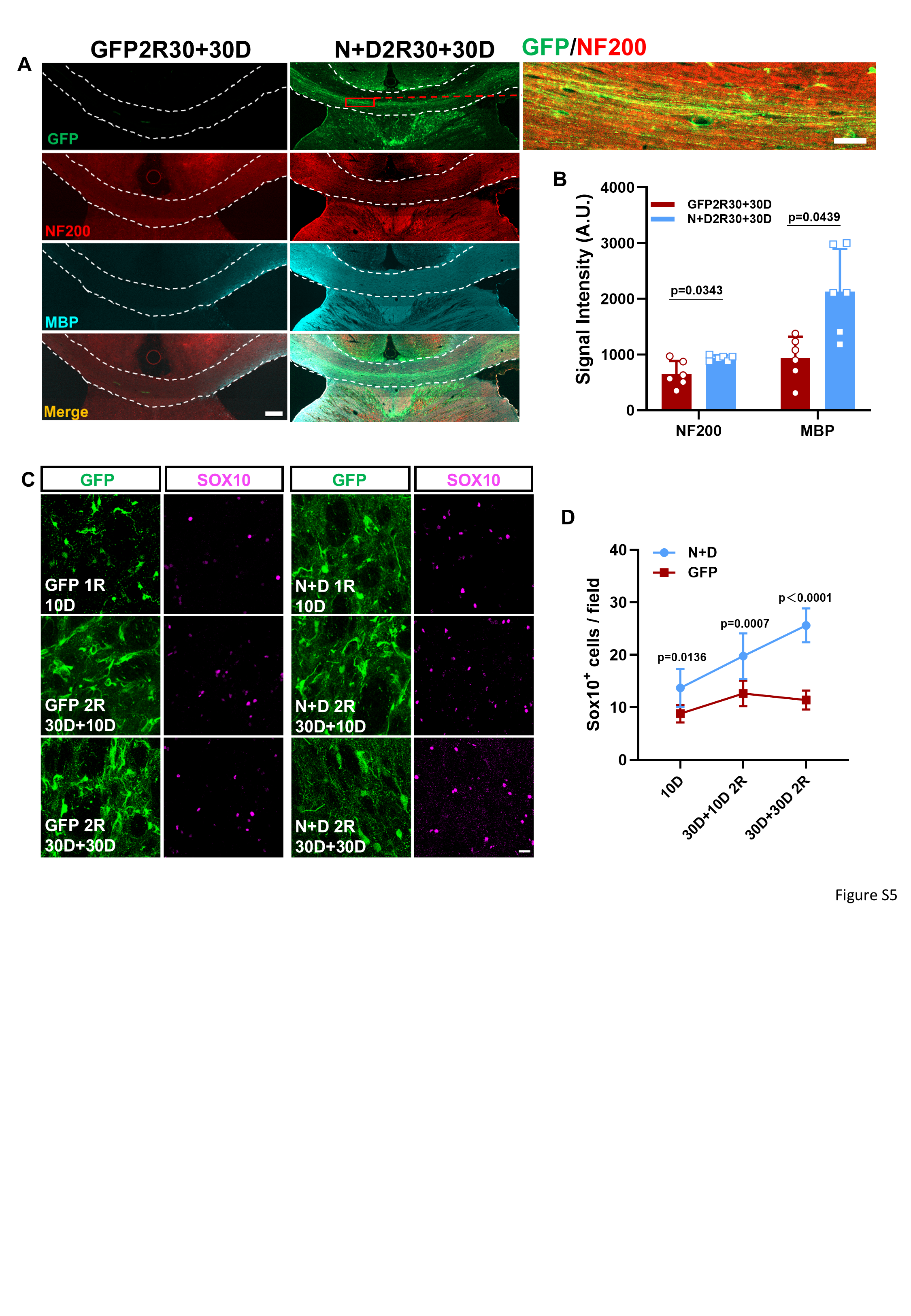

Supplement: Supplementary file 5 — Figure S5 (related to Figure 6). White matter repair achieved by AtN conversion. [file CNS-31-e70448-s004.tif]
