# Supplementary material for: Transcription Factor‐Based Gene Therapy Enables Functional Repair of Rat Following Chronic Ischemic Stroke
Source: CNS Neurosci Ther. 2025 May 22;31(5):e70448. doi: 10.1111/cns.70448 (PMC12096174; doi:10.1111/cns.70448)
Supplement: Supplementary file 6 — Table S1 Primary antibodies. [file CNS-31-e70448-s006.docx]

**TableS1**

**Primary antibodies**

| **Antibodies** | **Species** | **Catalog** | **Source** | **Dilution** |
| --- | --- | --- | --- | --- |
| NEUN | Rabbit | Ab177487 | Abcam | 1:500 |
| NEUN | Guinea pig | ABN90 | Millipore | 1:1500 |
| NEUROD1 | Rabbit | ab205300 | Abcam | 1:500 |
| GFP | Mouse | A11120 | Abcam | 1:1000 |
| GFP | Chicken | Ab13970 | Abcam | 1:1000 |
| GFP | Rabbit | A11122 | Invitrogen | 1:1000 |
| IBA1 | Rabbit | 019-19741 | Wako | 1:1000 |
| AQP4 | Rabbit | 16473-1-AP | Proteintech | 1:1000 |
| CD31 | Rat | 550274 | BD Bioscience | 1:300 |
| GFAP | Rabbit | G9269 | Sigma | 1:500 |
| GFAP | Rat | 13-0300 | Invitrogen | 1:1000 |
| OLIG2 | Rabbit | AB9610 | Millipore | 1:500 |
| OLIG2 | Mouse | MABN50 | Millipore | 1:500 |
| PV | Mouse | 235 | Swant | 1:1000 |
| DLX2 | Rabbit | Ab135620 | Abcam | 1:500 |
| DARPP32 | Rabbit | 2306S | CST | 1:1000 |
| CTIP2 | Rat | Ab18465 | Abcam | 1:500 |
| CSPG | Mouse | C8035 | Sigma | 1:1000 |
| APC | Mouse | Ab16794 | Abcam | 1:1000 |
| SST | Rat | MAB354 | Millipore | 1:1000 |
| SATB2 | Guinea pig | 327004 | SYSY | 1:500 |
| TBR1 | Rabbit | Ab31940 | Abcam | 1:1000 |
| NOS | Rabbit | N7280 | Sigma | 1:1000 |
| CUX1 | Rabbit | OB-PRB038 | Oasisbiofarm | 1:1000 |
| LAMININ | Rabbit | L9393 | Sigma | 1:1000 |
| NG2 | Mouse | MAB5384 | Millipore | 1:500 |
| NF200 | Mouse | N0142 | Sigma | 1:500 |
| MBP | Rabbit | Ab40390 | Abcam | 1:500 |
| SOX10 | Rabbit | Ab227680 | Abcam | 1:1000 |
| DAPI | - | 70508621 | Roche | 0.5 μg/mL |

**Secondary antibodies**

| **Antibodies** | **Species** | **Catalog** | **Source** | **Dilution** |
| --- | --- | --- | --- | --- |
| Anti-rat 488 | donkey | A21208 | Life Technologies | 1:1000 |
| Anti-rabbit 488 | donkey | A21206 | Life Technologies | 1:1000 |
| Anti-mouse 488 | donkey | A21202 | Life Technologies | 1:1000 |
| Anti-rabbit 555 | donkey | A31572 | Life Technologies | 1:1000 |
| Anti-mouse 555 | donkey | A31570 | Life Technologies | 1:1000 |
| Anti-rat 555 | donkey | 712-165-150 | Jackson immune research | 1:1000 |
| Anti-rabbit 594 | donkey | A21207 | Life Technologies | 1:1000 |
| Anti-mouse 647 | donkey | A31571 | Life Technologies | 1:500 |
| Anti-rabbit 647 | donkey | 711-605-152 | Jackson immune research | 1:500 |
| Anti-guinea pig 647 | donkey | 706-605-148 | Jackson immune research | 1:500 |
